# The Role of Individual Domains and the Significance of Shedding of ATP6AP2/(pro)renin Receptor in Vacuolar H^+^-ATPase Biogenesis

**DOI:** 10.1371/journal.pone.0078603

**Published:** 2013-11-04

**Authors:** Kenichiro Kinouchi, Atsuhiro Ichihara, Motoaki Sano, Ge-Hong Sun-Wada, Yoh Wada, Hiroki Ochi, Toru Fukuda, Kanako Bokuda, Hideaki Kurosawa, Naohiro Yoshida, Shu Takeda, Keiichi Fukuda, Hiroshi Itoh

**Affiliations:** 1 Division of Endocrinology, Metabolism, and Nephrology, Department of Internal Medicine, Keio University School of Medicine, Tokyo, Japan; 2 Department of Endocrinology and Hypertension, Tokyo Women’s Medical University, Tokyo, Japan; 3 Department of Cardiology, Keio University School of Medicine, Tokyo, Japan; 4 Department of Biochemistry, Faculty of Pharmaceutical Sciences, Doshisha Women’s College, Kyoto, Japan; 5 Division of Biological Sciences, Institute of Scientific and Industrial Research, Osaka University, Osaka, Japan; John Hunter Hospital, Australia

## Abstract

The ATPase 6 accessory protein 2 (ATP6AP2)/(pro)renin receptor (PRR) is essential for the biogenesis of active vacuolar H^+^-ATPase (V-ATPase). Genetic deletion of ATP6AP2/PRR causes V-ATPase dysfunction and compromises vesicular acidification. Here, we characterized the domains of ATP6AP2/PRR involved in active V-ATPase biogenesis. Three forms of ATP6AP2/PRR were found intracellularly: full-length protein and the N- and C-terminal fragments of furin cleavage products, with the N-terminal fragment secreted extracellularly. Genetic deletion of ATP6AP2/PRR did not affect the protein stability of V-ATPase subunits. The extracellular domain (ECD) and transmembrane domain (TM) of ATP6AP2/PRR were indispensable for the biogenesis of active V-ATPase. A deletion mutant of ATP6AP2/PRR, which lacks exon 4-encoded amino acids inside the ECD (Δ4M) and causes X-linked mental retardation Hedera type (MRXSH) and X-linked parkinsonism with spasticity (XPDS) in humans, was defective as a V-ATPase-associated protein. Prorenin had no effect on the biogenesis of active V-ATPase. The cleavage of ATP6AP2/PRR by furin seemed also dispensable for the biogenesis of active V-ATPase. We conclude that the N-terminal ECD of ATP6AP2/PRR, which is also involved in binding to prorenin or renin, is required for the biogenesis of active V-ATPase. The V-ATPase assembly occurs prior to its delivery to the *trans*-Golgi network and hence shedding of ATP6AP2/PRR would not affect the biogenesis of active V-ATPase.

## Introduction

Binding of prorenin to the (pro)renin receptor (PRR) not only generates local angiotensin I due to the non-proteolytic activation of prorenin, but also induces intracellular signaling [1]. We have shown that PRR activation is involved in the development of cardiac fibrosis and proteinuria in hypertension and diabetes [2]–[6].

PRR consists of four different domains: signal peptide (SP), extracellular domain (ECD), transmembrane domain (TM), and cytosolic domain (CD). Under physiological conditions, PRR is cleaved in the trans-Golgi network by the protease furin, generating an amino-terminal fragment (NTF) and a carboxyl-terminal fragment (CTF) [7]. The NTF binds to prorenin, even after cleavage, and is excreted from the cell [8].

Prior to the cloning of PRR, the encoding gene was named ATP6ap2 (ATPase 6 accessory protein 2) due to the demonstrated association of part of the CTF, termed M8.9, with the vacuolar H^+^-ATPase (V-ATPase) [9]. We and other groups recently demonstrated that ATP6AP2/PRR is essential for the biogenesis of active V-ATPase [10]–[12]. The tissue expression levels of ATP6AP2/PRR are also closely correlated with other V-ATPase subunits in mice, implicating an essential function for ATP6AP2/PRR as a mammalian V-ATPase-related protein [13].

It therefore could be speculated that the CTF and NTF have separable functions in terms of PRR and its function as an accessory protein of V-ATPase, respectively. However, a recent study suggested that the ECD, part of which NTF resides, is also required for ATP6AP2/PRR to bind with the V-ATPase subunits [14]. Thus, the role of each domain of ATP6AP2/PRR and the significance of shedding events with regard to active V-ATPase biogenesis remain to be clarified.

Mutation in the *ATP6AP2/PRR* gene (MIM 300556) is a cause of X-linked mental retardation Hedera type (MRXSH) (OMIM # 300423) as well as the newly reported X-linked parkinsonism with spasticity (XPDS) syndrome in humans [15], [16]. Affected patients have a single mutation in the exon-splicing enhancer site of ATP6AP2/PRR, which results in a shorter ATP6AP2/PRR fragment with a deletion of exon 4 (Δ4M). The exon 4-encoded amino acids are localized in the middle part of the ECD. Previous studies demonstrated that human Δ4M could bind renin and increase renin catalytic activity, similarly to full-length ATP6AP2/PRR, but that it could also induce a modest and reproducible impairment of prorenin- and renin-induced extracellular signal-related protein kinase (ERK) 1/2 activation [16], [17]. However, no studies have investigated the impact of human Δ4M with regard to V-ATPase activities.

The present study thus aimed to characterize the domain-specific functions and significance of shedding for ATP6AP2/PRR in association with V-ATPase.

## Materials and Methods

### Animals

All animal experiments were reviewed and approved by the Institutional Animal Care and Use Committee at Keio University School of Medicine.

### Expression constructs

Mouse and human ATP6AP2/PRR cDNA was obtained from NCBI (accession: NM_027439.4 and NM_005765.2, respectively). Tagged mouse ATP6AP2/PRR constructs were generated by inserting ATP6AP2/PRR into a pMXs-IG (IRES-GFP)-based vector to yield a C-terminal Flag tag [18], [19]. The ATP6AP2/PRR mutants originated from a mouse cDNA library: CTF mutants lack aa 18–275 of the extracellular domain; ΔCD mutants, aa 335–350 of the cytosolic domain; NTF mutants, aa 276–350 of the extracellular, transmembrane, and cytosolic domains, respectively. ATP6AP2/PRR mutated in the potential furin cleavage site was created by mutagenesis of the R276A/KT/R279A site. The human ATP6AP2/PRR constructs were generated as for mouse, and then inserted into a pMXs-IG vector with a C-terminal Flag tag. Human Δexon 4 mutant (Δ4M) lacks aa 101-132. Mouse furin cDNA was obtained from NCBI (accession: NM_011046), and mouse furin constructs were generated by inserting furin into a pMXs-IG-based vector.

### Retrovirus production

Retrovirus was generated according to the protocol reported previously [20]. Briefly, pMXs-IG retroviral plasmid DNA encoding each ATP6AP2/PRR motif and furin was transfected into Plat-E packaging cells. Two days later, the Plat-E cell medium containing virus was collected and added to mouse embryonic fibroblast (MEF) cultures.

### Cell culture

MEFs were obtained from male mouse embryos. ATP6AP2/PRR floxed MEFs were hemizygous for the floxed *Atp6ap2* allele. MEFs were maintained in Dulbecco’s modified Eagle’s medium (DMEM; Invitrogen, Carlsbad, CA, USA) containing 10% fetal bovine serum (FBS; Invitrogen, Carlsbad, CA, USA) and cultured at 37°C in 10% CO_2_. MEFs were treated with 10 µM cycloheximide (Wako Pure Chemical Industries, Osaka, Japan) to inhibit de novo protein synthesis, and with 5 µM MG132 (Sigma, St. Louis, MO, USA) to inhibit the degradation of ubiquitin-conjugated proteins.

### Prorenin preparation and incubation

For analysis of ERK signaling, the cells were conditioned for 24 h with the serum-free medium, and then treated with 2 nM human recombinant prorenin (Cayman chemical company, Ann Arbor, MI, USA) for 60 minutes. The cells were then harvested for immunoblotting.

### Antibodies

Antibodies against V-ATPase subunits and Rab7 were as described previously [21]–[24]. Other primary antibodies used were monoclonal antibodies against Lamp2 (DSHB, Iowa City, Iowa, USA), GM130, BiP/GRP78 (BD Biosciences, San Jose, CA, USA), early endosomal antigen 1 (EEA1, C-terminal; Sigma, St. Louis, MO, USA), and GAPDH (Cell Signaling Technology, Danvers, MA, USA), as well as polyclonal antibodies against ATP6AP2/PRR (R&D Systems, Minneapolis, MN, USA), Rab5 (Enzo Life Sciences, Farmingdale, NY, USA), phosphorylated ERK, ERK (Cell Signaling Technology, Danvers, MA, USA), and LC3 (a gift from Dr. Komatsu, Tokyo Metropolitan Institute of Medical Science). Fluorochrome- or enzyme-linked secondary antibodies were obtained from Invitrogen (Carlsbad, CA, USA).

### Western blotting

Cell layers were extracted with RIPA buffer containing 50 mM Tris-HCl Buffer (pH 7.6), 150 mM NaCl, 1% Nonidet P40, 0.5% sodium deoxycholate, 0.1% SDS, and protease inhibitor cocktail (Nacalai Tesque, Kyoto, Japan). Total lysates were applied (10–20 µg/lane) to Any kD™ Mini-PROTEAN® TGX™ Precast Gel (Bio-Rad, Hercules, CA, USA) and then transferred to nitrocellulose membrane (Bio-Rad, Hercules, CA, USA). Membranes were blocked with Blocking One (Nacalai Tesque, Kyoto, Japan) for 1 hour at RT. Subsequently, immunoblots were incubated with the aforementioned antibodies. Protein expression was visualized using a horseradish peroxidise (HP)–conjugated secondary antibody and enhanced chemiluminescence (Nacalai Tesque, Kyoto, Japan), and images obtained using an image capture system (model LAS3000 luminoimager; Fujifilm, Tokyo, JAPAN) were quantified for band intensities using Image Gauge Software (Fujifilm) and Image J. All experiments were repeated at least three times. β-actin was used as a loading control, and molecular weight was calibrated using Precision Plus Protein Dual Color Standards (Bio-Rad, Hercules, CA, USA).

### siRNA oligonucleotides and transfection

siRNA oligonucleotides against the *furin* gene, as well as non-targeting siRNA as a vehicle treated control, were purchased from Thermo Fisher Scientific (Lafayette, CO, USA). Transfection of these siRNA oligonucleotides was achieved using Lipofectamine™ 2000 reagent (Invitrogen, Carlsbad, CA, USA).

### LysoTracker analysis

The immunofluorescence of cultured cells was conducted as described previously [25]. To label acidic organelles, cells were incubated with LysoTracker (Invitrogen, Carlsbad, CA, USA) for 30 min, before being fixed with 4% paraformaldehyde in phosphate-buffered saline (pH 7.4).

### Confocal microscopy

Immunostained and transfected cells were analyzed as described previously [25]. MEFs were observed 3 days after the transfection of mutant ATP6AP2/PRR by confocal microscopy (Carl Zeiss, Oberkochen, Germany).

### Statistics

Data are presented as mean ± SD. Distribution was evaluated by the *F*-test. The significance of differences between two mean values was evaluated by Student’s *t*-test. Analyses are two-sided, with a *P* value of 0.05 or less considered to indicate statistical significance. The level of colocalization between ATP6AP2/PRR-FLAG and each subcellular compartment was determined by Mander’s coefficients for colocalization. Half-lives were determined by approximated exponent curves. Analyses were performed using Microsoft Office Excel 2007, Image J, and StatView 5.0 software (SAS Institute Inc., Cary, NC, USA).

## Results

### ATP6AP2/PRR exists as the full-length protein and two furin cleavage products, the C- and N-terminal fragments

**Figure 1:**
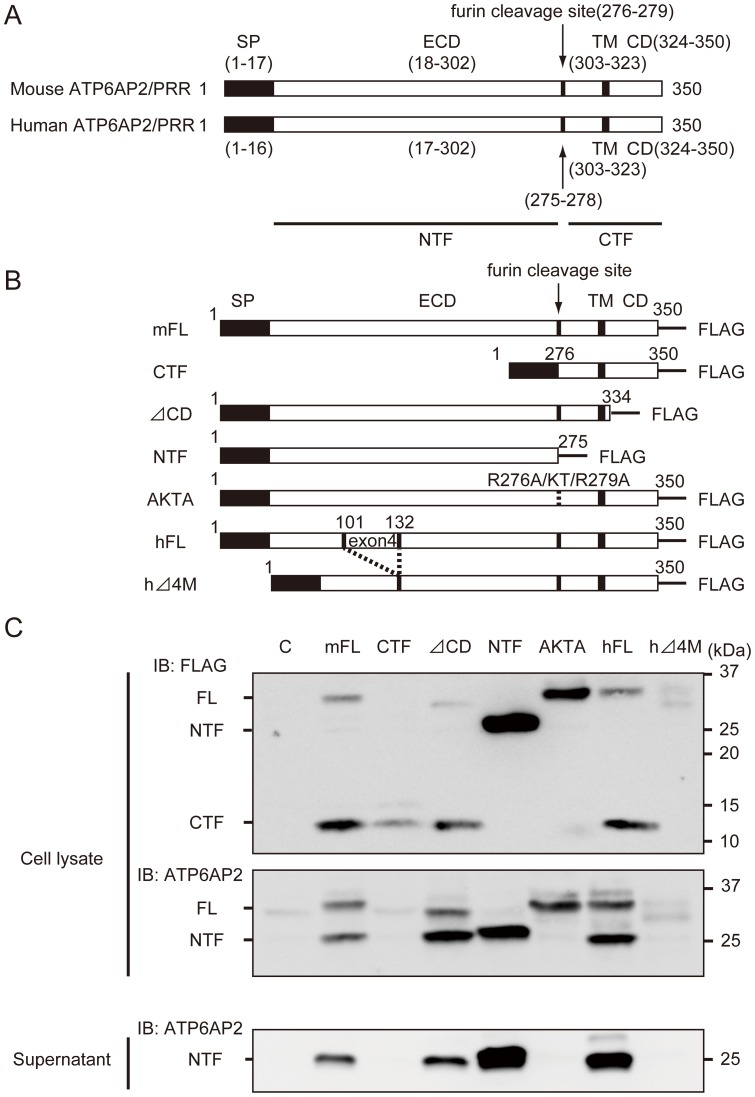
ATP6AP2/(pro)renin receptor (PRR) existed in three forms, the full-length protein and the N- and C-terminal fragment of furin cleavage products, and the N-terminal fragment is secreted extracellularly. **A**, Schematic representation of mouse and human ATP6AP2/PRR structures. ATP6AP2/PRR comprises four different domains: signal peptide (SP), extracellular domain (ECD), transmembrane domain (TM), and cytosolic domain (CD). A portion of ATP6AP2/PRR is cleaved in the trans-Golgi network by the protease furin, generating an amino-terminal fragment (NTF) and a carboxyl-terminal fragment (CTF). **B**, Constructed retroviral vectors harboring full-length mouse and human ATP6AP2/PRR or one of several deletion mutants of mouse and human ATP6AP2/PRR; CTF, ΔCD, NTF, AKTA (mutation within the putative furin recognition site), and Δ4M (a shorter ATP6AP2/PRR fragment with a deletion of exon 4). **C**, The full-length ATP6AP2/PRR and its mutants were expressed in mouse embryonic fibroblasts (MEFs). Anti-FLAG antibody detected a FLAG-tagged exogenous protein, whereas an anti-ATP6AP2/PRR antibody designed to recognize the N-terminal extracellular domain (ECD) of ATP6AP2/PRR detected both endogenous ATP6AP2/PRR and exogenous mutant protein containing ECD. Exogenously expressed ATP6AP2/PRR was detected in three forms: full-length protein and the N- and C-terminal products of furin cleavage. The NTF is secreted extracellularly, suggesting that a significant amount of exogenous ATP6AP2/PRR is cleaved intracellularly. The gene products of AKTA were resistant to furin cleavage, as expected. SP, signal peptide; ECD, extracellular domain; TM, transmembrane domain; CD, cytoplasmic domain; FL, full-length ATP6AP2/PRR; CTF, carboxyl-terminal fragment; NTF, amino-terminal fragment; AKTA, ATP6AP2/PRR mutated within the furin recognition site R276A/KT/R279A; Δ4M, ATP6AP2/PRR with a deletion of exon 4; C, control.

We systematically constructed a series of retroviral vectors harboring full-length mouse ATP6AP2/PRR or one of the following deletion mutants: CTF, ΔCD, NTF, and R276A/KT/R279A (AKTA, mutation within the putative furin recognition site), human full-length ATP6AP2/PRR, and human Δ4M (Figure 1A, B). We tagged the C-terminal portion of each construct with a FLAG epitope and expressed them in cultured MEFs. Anti-FLAG antibody detected a FLAG-tagged exogenous protein, whereas anti-ATP6AP2/PRR antibody, designed to recognize the N-terminal ECD of ATP6AP2/PRR, detected both endogenous ATP6AP2/PRR and exogenous mutant protein containing ECD (Figure 1C). Exogenously expressed full-length ATP6AP2/PRR was detected in three forms, the full-length protein and the N- and C-terminal fragments produced by furin cleavage. NTF is secreted extracellularly, suggesting that a significant amount of ATP6AP2/PRR is cleaved intracellularly. Endogenous ATP6AP2/PRR was less cleaved in MEFs compared to exogenous ATP6AP2/PRR (Figure 1C, S1A). Exogenously expressed mouse and human full-length ATP6AP2/PRR were equally processed (Figure S1B, FL/NTF, 2.5 ± 0.7 vs 2.2 ± 0.1 for mouse vs. human; *P* = 0.46). The five mouse mutant and two human mutant ATP6AP2/PRR constructs yielded an expressed protein of identical size, and the gene products of AKTA were resistant to furin cleavage, as expected (Figure 1C). Interestingly, human Δ4M also appeared resistant to cleavage despite preserving the putative furin recognition site, based on the absence of NTF in the supernatant (Figure 1C).

To test the stability of the respective mutant ATP6AP2/PRR, MEFs were treated with cycloheximide to inhibit de novo protein synthesis 72 h after transfection with FLAG-ATP6AP2/PRR and observed at various time points up to 6 h. The signal intensity of newly synthesized exogenous ATP6AP2/PRR as well as endogenous ATP6AP2/PRR rapidly decreased by 6 h after cycloheximide treatment (Figure S1A, S2A), strongly indicating the requirement of ongoing protein synthesis to maintain steady-state levels of ATP6AP2/PRR protein. Half-lives of each mutant ATP6AP2/PRR ranged from 1 to 3 hours (Figure S2B). Consistent with the previous findings, human Δ4M seemed especially unstable, since the signal density was too low from the first time point to calculate the exact half-life when blotted with FLAG [16], [17]. Proteasome inhibition with MG132 partially inhibited the respective decreases in protein expression, suggesting that exogenous ATP6AP2/PRR is partly degraded via the ubiquitin-proteasome system.

Next, we investigated the intracellular localization of exogenously expressed full-length and mutated ATP6AP2/PRR by double immunostaining with anti-FLAG antibody and markers for endoplasmic reticulum (ER) (BiP), Golgi (GM130), early endosomes (EEA1, Rab5), late endosomes (Rab7), or lysosomes (LAMP2). Immunohistochemistry revealed that all mutants were distributed in a perinuclear pattern that partially colocalized with each subcellular compartment, although none of them were solely localized in any specific organelle (Figure S3-S8). The ATP6AP2/PRR mutants also showed partial colocalization with ATP6V0C (V-ATPase subunit c) antibody staining (Figure S9), although we could not specify the intracellular compartment where ATP6AP2/PRR-FLAG meets the V-ATPase.

### The extracellular and transmembrane domains of ATP6AP2/PRR are essential for the biogenesis of active V-ATPase

**Figure 2:**
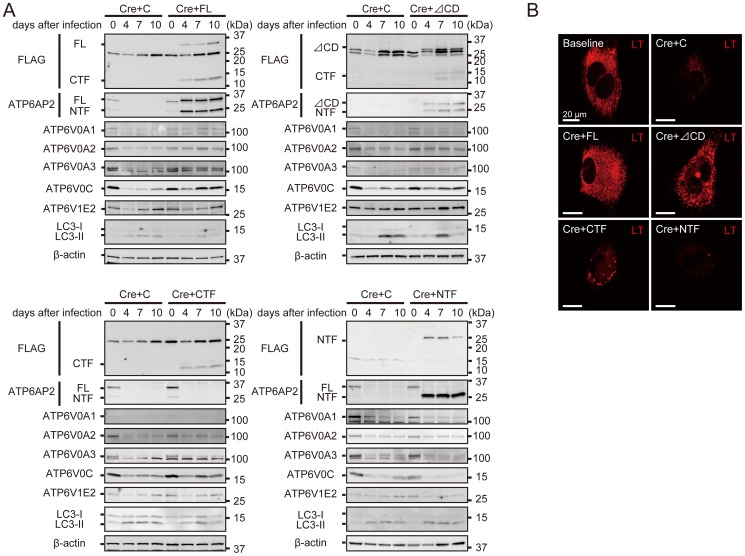
The extracellular domain (ECD) and transmembrane domain (TM) were indispensable for the biogenesis of active V-ATPase. **A**, *Atp6ap2*-floxed MEFs were treated with Cre-adenovirus and ATP6AP2/(pro)renin receptor (PRR) mutant retrovirus. The levels of V_O_ subunits decreased significantly in the floxed MEFs after deletion of the ATP6AP2/PRR, whereas the V_1_ subunit ATP6V1E2 seemed unaffected. Note that LC3-II expressions were upregulated after the ATP6AP2/PRR deletion. These changes were rescued by either exogenously expressed full-length ATP6AP2/PRR (FL) or ΔCD, while exogenous expression of the carboxyl-terminal fragment (CTF) or the amino-terminal fragment (NTF) failed to abrogate these changes. Note that CTF partly restored the expression of ATP6V0A3 and ATP6V0C, and reduced LC3-II accumulation. The bands at ∼25 kDa, which are visible in the blots probed with anti-FLAG polyclonal antibody when FL, ΔCD, or CTF was exogenously expressed, are non-specific bands. NTF was probed with anti-FLAG monoclonal antibody to avoid overlapping with non-specific bands. **B**, LysoTracker staining demonstrated the compromised intravesicular acidification in the floxed MEFs after Cre-adenovirus infection. FL and ΔCD reversed the defective intravesicular acidification in the floxed MEFs after deletion of ATP6AP2/PRR, while CTF- and NTF-expressing cells remained defective. Scale bars, 20 µm. C, empty control retroviral vector (pMXs-IG); FL, full-length ATP6AP2/PRR; CTF, carboxyl-terminal fragment; CD, cytoplasmic domain; NTF, amino-terminal fragment; LT, LysoTracker.

MEFs obtained from male mice hemizygous for the floxed *Atp6ap2* allele [10] were infected with the Cre adenovirus (Ad-Cre) for *Atp6ap2* gene ablation (Figure 2A). Western blot analyses showed that ≥ 90% of the ATP6AP2/PRR protein was deleted in the floxed MEFs after Ad-Cre treatment. V-ATPase is a large multi-subunit complex organized into V_1_ and V_O_ sectors. In mammals, the V_1_ sector is composed of eight different subunits, whereas the V_O_ sector contains six different subunits [26]. Western blot analyses revealed that the levels of V_O_ subunits ATP6V0A1, ATP6V0A2, ATP6V0A3, and ATP6V0C were significantly decreased in the floxed MEFs after Ad-Cre infection, but V_1_ subunit ATP6V1E2 was unaffected. LC3-II enhancement occurs as a consequence of impaired autophagic degradation. Consistent with these findings, LysoTracker staining revealed that the loss of ATP6AP2/PRR was accompanied by impaired vesicular acidification (Figure 2B). To study the impact of ATP6AP2/PRR on the protein stability of V-ATPase subunits, MEFs were subjected to cycloheximide treatment in the presence or absence of ATP6AP2/PRR. V-ATPase subunits were stable in wild-type MEFs, since the treatment of cycloheximide had no effect on the expression levels of any of the V-ATPase subunits (Figure S1A). Notably, the protein turnover of these subunits stayed unaffected even after ATP6AP2/PRR removal.

Exogenously expressed full-length or ΔCD restored the expression levels of Vo subunits, LC3-II accumulation, and intracellular vesicular acidification in ATP6AP2/PRR-ablated MEFs, although ΔCD did not restore the expression levels of ATP6V0A1 and ATP6V0A2 significantly (the level of restoration of LysoTracker staining from the baseline; full-length, 119%; ΔCD, 79%). By contrast, exogenously expressed CTF or NTF failed to rescue the phenotype observed with the ablation of endogenous ATP6AP2/PRR, although CTF seemed to achieve partial rescue (the level of restoration of LysoTracker staining from the baseline; CTF, 38%; NTF, 14%) (Figure 2A-B, 3A-D). The bands at ∼25 kDa, which are visible in the blots probed with anti-FLAG polyclonal antibody when full-length, ΔCD, or CTF was exogenously expressed, are non-specific bands. NTF was thus probed with anti-FLAG monoclonal antibody to avoid overlapping with non-specific bands.

**Figure 3:**
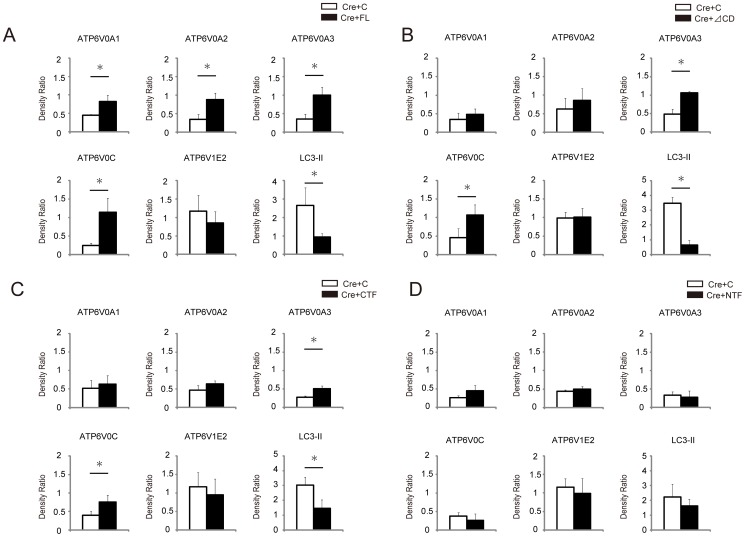
Quantitative analysis of protein expressions of V-ATPase subunits, ATP6V0A1, ATP6V0A2, ATP6V0A3, ATP6V0C, and ATP6V1E2, as well as autophagosomal marker LC3-II in MEFs after infection with Cre-adenovirus and retroviral transgenic overexpression either with empty control (C), full-length ATP6AP2/PRR (FL) (A), cytosolic domain-deleted ATP6AP2/PRR (ΔCD) (B), the carboxyl-terminal fragment of ATP6AP2/PRR (CTF) (C), or the amino-terminal fragment of ATP6AP2/PRR (NTF) (D). The levels of V_O_ subunits ATP6V0A1, ATP6V0A2, ATP6V0A3, and ATP6V0C were decreased in the floxed MEFs after Ad-Cre infection, but V_1_ subunit ATP6V1E2 was unaffected. LC3-II accumulation occurs as a consequence of impaired autophagic degradation. Exogenously expressed FL or ΔCD restored the expression levels of Vo subunits and LC3-II accumulation in ATP6AP2/PRR-ablated MEFs. By contrast, exogenously expressed CTF or NTF failed to rescue the phenotypes. CTF partly restored the expression of ATP6V0A3 and ATP6V0C, and reduced LC3-II accumulation. The data are presented as the ratio of protein expressions in day 10 to those in day 0. Graphed data show the mean ± SD. * *P* < 0.05 compared with control retrovirus. C, empty control retroviral vector (pMXs-IG); FL, full-length ATP6AP2/PRR; CTF, carboxyl-terminal fragment; CD, cytoplasmic domain; NTF, amino-terminal fragment.

### Human Δ4M is defective for an accessory protein of V-ATPase

Next, we examined the function of human Δ4M, which is observed in families with MRXSH and XPDS, with regard to active V-ATPase biogenesis. The exon 4-encoded amino acids are localized in the middle part of the ECD and the extensive deletion of ECD extinguished the ability of ATP6AP2/PRR to participate in active V-ATPase biogenesis, although lack of TM and CD also failed to rescue V-ATPase (Figure 2).

**Figure 4:**
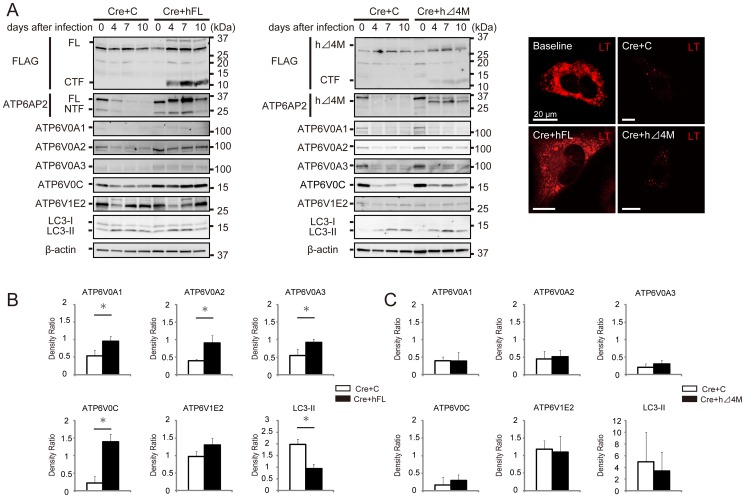
Human Δ4M is defective as an accessory protein of V-ATPase. **A**, The human full-length ATP6AP2/PRR (hFL) or human Δ4M was transgenically overexpressed in ATP6AP2/PRR-deficient MEFs. The hFL restored the expression levels of Vo subunits, LC3-II accumulation, and vesicular acidification similar to those observed with the mouse full-sized ATP6AP2/PRR. By contrast, human Δ4M could not rescue the phenotype observed for ATP6AP2/PRR-deficient MEFs. Scale bars, 20 µm. **B, C,** Quantitative analysis of protein expressions of ATP6V0A1, ATP6V0A2, ATP6V0A3, ATP6V0C, ATP6V1E2, and LC3-II in MEFs after transgenic overexpression of human mutant ATP6AP2/PRR. *Atp6ap2*-floxed MEFs were treated with Cre-adenovirus and retroviral transgenic overexpression with empty control (C), hFL (**B**), or human Δ4M (**C**). The hFL rescued the Vo subunit expressions and LC3-II accumulation observed for ATP6AP2/PRR-deficient MEFs, while human Δ4M failed to restore these phynotypes. The data are presented as the ratio of protein expressions in day 10 to those in day 0. Graphed data show the mean ± SD. * *P* < 0.05 compared with transgenic overexpression with control retrovirus. C, empty control retroviral vector (pMXs-IG); FL, full-length ATP6AP2/PRR; CTF, carboxyl-terminal fragment; NTF, amino-terminal fragment; Δ4M, ATP6AP2/PRR with a deletion of exon 4; LT, LysoTracker.

The human full-length ATP6AP2/PRR or human Δ4M were then transgenically overexpressed in ATP6AP2/PRR-deficient MEFs. The human full-length protein could restore the expression levels of Vo subunits, LC3-II accumulation, and vesicular acidification, similarly to mouse full-length ATP6AP2/PRR. By contrast, human Δ4M could not rescue the phenotype observed for ATP6AP2/PRR-deficient MEFs, suggesting that exon 4-encoded amino acids localized in the middle part of the ECD is a prerequisite for ATP6AP2/PRR to function as an accessory protein of V-ATPase (the level of restoration of LysoTracker staining from the baseline; full-length, 89%; Δ4M, 12%) (Figure 4A-C).

### Binding of prorenin to ATP6AP2/PRR did not affect active V-ATPase biogenesis

Since the extracellular domain of ATP6AP2/PRR is also essential for binding of renin or prorenin [8], we tested the effect of prorenin on active V-ATPase biogenesis. Binding of prorenin to ATP6AP2/PRR mediates angiotensin II formation and activates both ERK1/2 and p38 pathways [27], although whether binding of prorenin to ATP6AP2/PRR modulates the biogenesis of active V-ATPase remains unknown. To address this question, MEFs were stimulated with 2 nM of prorenin. Although prorenin stimulation increased the phosphorylation levels of ERK1/2 (Figure S10A), it did not affect the expression levels of any V-ATPase subunits or the acidification of intracellular vesicular compartments as shown by LysoTracker staining (Figure S10A-C).

### The cleavage of ATP6AP2/PRR by furin seems to be dispensable for active V-ATPase biogenesis

**Figure 5:**
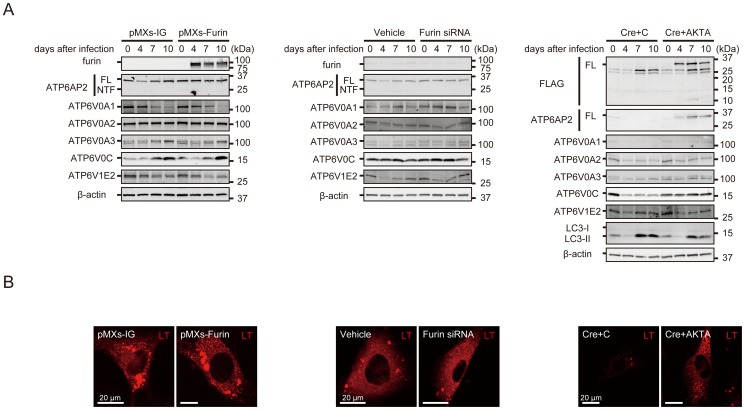
The cleavage of ATP6AP2/(pro)renin receptor (PRR) by furin seems to be dispensable for active V-ATPase biogenesis. **A**, Wild-type MEFs were treated by transgenic overexpression of furin or furin siRNA-mediated suppression. Both overexpression and siRNA-mediated suppression of furin had no effect on the expressions of V-ATPase subunits. Furthermore, *Atp6ap2*-floxed MEFs were subject to Cre-adenovirus and retroviral transgenic overexpression with either empty control (C) or ATP6AP2/PRR mutated in the potential furin cleavage R276A/KT/R279A (AKTA) site. AKTA successfully recovered the expression of ATP6V0A3 and ATP6V0C and abrogated the LC3-II expression, although AKTA did not restore the expression levels of ATP6V0A1 and ATP6V0A2. **B**, LysoTracker staining demonstrated that furin overexpression or downregulation also had no effect on intravesicular acidification, and AKTA recovered the defective acidification in the floxed MEFs after deletion of ATP6AP2/PRR. Scale bars, 20 µm. pMXs-IG, pMXs-IRES-GFP; FL, full-length ATP6AP2/PRR; NTF, amino-terminal fragment; C, empty control retroviral vector (pMXs-IG); AKTA, ATP6AP2/PRR mutated in the potential furin cleavage R276A/KT/R279A site; LT, LysoTracker.

Lastly, we examined whether shedding of ATP6AP2/PRR by furin affects the biogenesis of active V-ATPase. Neither overexpression nor siRNA-mediated knockdown of furin influenced the protein expression of V-ATPase subunits or endoluminal acidification as shown by LysoTracker staining (Figure 5, 6A, B). Intriguingly, overexpression of furin also did not increase the shedding of ATP6AP2/PRR; however, suppression of furin seemed to somewhat reduce the processing of ATP6AP2/PRR, although very little endogenous ATP6AP2/PRR is cleaved to NTF in MEFs (Figure 5A, B). Furthermore, transgenic rescue of ATP6AP2/PRR-null MEFs with furin-insensitive ATP6AP2/PRR mutant AKTA restored the expression levels of ATP6V0A3, ATP6V0C, LC3-II accumulation, and vesicular acidification, although AKTA did not restore the expression levels of ATP6V0A1 and ATP6V0A2 (Figure 5A and 5B, 6C). Taken together, these findings indicated that the cleavage of ATP6AP2/PRR by furin seems to be dispensable for active V-ATPase biogenesis.

**Figure 6:**
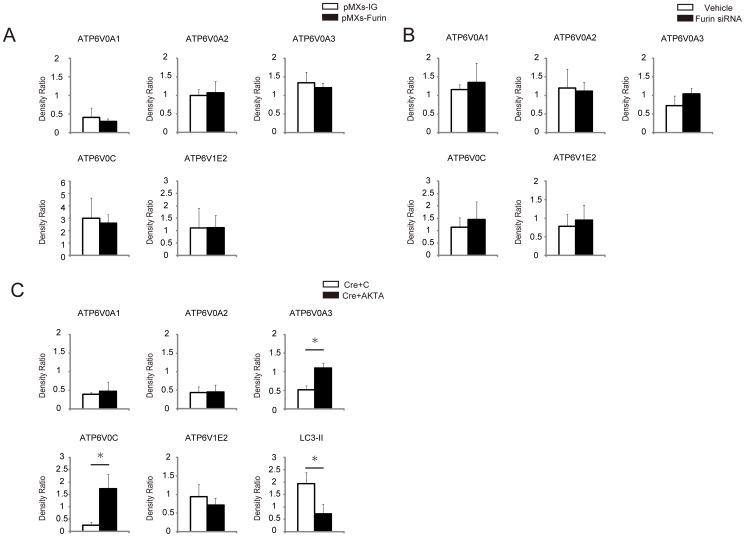
Quantitative analysis of protein expressions of ATP6V0A1, ATP6V0A2, ATP6V0A3, ATP6V0C, ATP6V1E2, and LC3-II in wild-type MEFs after transgenic overexpression or downregulation of furin (A, B) or in *Atp6ap2*-floxed MEFs treated with Cre-adenovirus and transgenic overexpression with control retrovirus (C) or ATP6AP2/PRR mutated in the potential furin cleavage R276A/KT/R279A (AKTA) site (C). Neither overexpression nor siRNA-mediated suppression of furin affected the expressions of V-ATPase subunits. AKTA successfully recovered the expressions of ATP6V0A3 and ATP6V0C and abrogated LC3-II expressions. The data are presented as the ratio of protein expressions in day 10 to those in day 0. Graphed data show the mean ± SD. * *P* < 0.05 compared with transgenic overexpression with empty control retroviral vector **(A, C)**, or non-targeting siRNA as a vehicle treated control **(B)**. pMXs-IG, pMXs-IRES-GFP; FL, full-length ATP6AP2/PRR; NTF, amino-terminal fragment; C, empty control retroviral vector (pMXs-IG); AKTA, ATP6AP2/PRR mutated in the potential furin cleavage R276A/KT/R279A site.

## Discussion

ATP6AP2/PRR is an accessory protein of V-ATPase and the V_O_ subunits of V-ATPase were detected at greatly reduced levels in mutant cells lacking ATP6AP2/PRR [10]. V_O_ subunits are assembled and delivered to the vacuole independently of V_1_ [28], and the assembly and stability of V_O_ subunits is severely compromised in the absence of any other V_O_ component. In yeast cells lacking the integral membrane portion of the V-ATPase complex, V_O_ subunits are not transported to and thus not stably associated with vacuolar membranes [29], [30]. V_O_ subunits were absent or present at a substantially reduced level in mutant vacuolar membrane fractions [31], [32]. Notably, the stability of the 100-kDa subunit, Vph1p, is drastically reduced in cells lacking other V_O_ subunits [33]. Genetic screens for yeast vacuolar membrane ATPase (vma) mutants have also led to the isolation of a set of dedicated chaperones residing in the ER that are required for V-ATPase biogenesis, but whose products are not structural components of V_O_V_1_ [34]. The biochemical phenotypes of vma12Δ, vma22Δ, and vma21Δ cells resemble those of strains lacking a structural subunit of V_O_. Interestingly, all three proteins localize to the ER, suggesting a dedicated role in V_O_ biogenesis [32], [34], [35].

In this study, the expression levels of newly synthesized V_O_ subunits were stable and their stability was not affected even after ATP6AP2/PRR removal. By contrast, ATP6AP2/PRR itself underwent more rapid turnover. ATP6AP2/PRR does not localize only to the ER, and only a portion of endogenous ATP6AP2/PRR is known to be processed in the TGN, since full-length ATP6AP2/PRR is still expressed at the cell surface where it can still bind to V_O_ [14]. We demonstrated in this study that cleavage of ATP6AP2/PRR by furin seems dispensable for V-ATPase regulation. Taken together, the results suggested that, at least in MEFs, most of the V_O_ would remain bound to full-length ATP6AP2/PRR after exit from the ER and this would be more reminiscent of a role in V-ATPase targeting for ATP6AP2/PRR instead of V-ATPase assembly. Future studies should clarify where in the cell ATP6AP2/PRR meets V-ATPase and how it associates with the V-ATPase complex.

**Figure 7:**
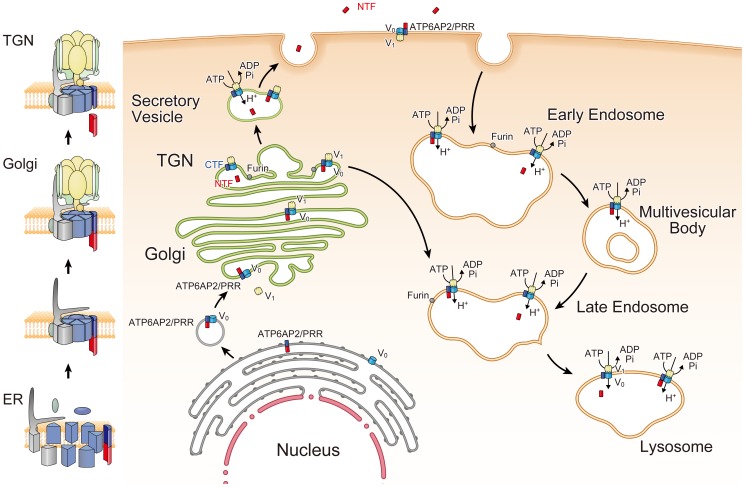
Overview of the intracellular dynamics of ATP6AP2/(pro)renin receptor (PRR) and V-ATPase. Full-length ATP6AP2/PRR is required for the V_O_ domain of V-ATPase. Fully assembled V_O_ domain in endoplasmic reticulum is transported to the Golgi where it combines with the V_1_ domain. In the trans-Golgi network, a portion of ATP6AP2/PRR undergoes cleavage by furin and the amino-terminal fragment of ATP6AP2/PRR (NTF) is secreted in vesicle to be excreted extracellularly via exocytosis, although this step seems unrelated to active V-ATPase biogenesis. FL, full-length ATP6AP2/PRR; CTF, carboxyl-terminal fragment; NTF, amino-terminal fragment; ER, endoplasmic reticulum; TGN, trans-Golgi network.

M8.9, which corresponds to a part of CTF, was originally reported to be associated with V-ATPase [9]. Sequential analysis demonstrated that M8.9 harbors the peptide “DPSTTYNLAYKYNF”, which resides in the ECD adjacent to the TM [9]. It is therefore reasonable to assume that the CTF is critically involved in the biogenesis of active V-ATPase. However, our study indicated that an exogenously expressed CTF could little rescue the reduced expression of V_O_ subunits or the impaired intravesicular acidification in ATP6AP2/PRR-deficient cells, but that TM and ECD could achieve such a rescue. This is consistent with the previous report that the TM and the ECD of ATP6AP2/PRR are required for binding ATP6V0C [14]. The CD of ATP6AP2/PRR is very short, comprising only 24 amino acids, but contains an endosomal and lysosomal sorting signal as well as ER retention motifs, suggesting that the CD controls targeting of the V-ATPase to specific subcellular organelles [36]. The cytoplasmic tail of another V-ATPase-associated protein, ATP6AP1/Ac45, was also found to be critical for the subcellular targeting of V-ATPase [37]. By contrast, ΔCD could rescue not only the expression of the V_O_ subunits of V-ATPase, but also the defective intravesicular acidification or the autophagy in ATP6AP2/PRR-deficient cells, although ΔCD did not restore the expression levels of ATP6V0A1 and ATP6V0A2 significantly. Since the *a*1 and *a*2 isoforms were localized to the Golgi apparatus or organelles other than lysosomes/late endosomes, the CD may play a role in targeting of the V-ATPase to subcellular organelles [38]. Then, what might be the role of M8.9/CTF that was originally found together with the Vo subunits from bovine chromaffin granules [9]? The biogenesis of the multi-subunit complex of V-ATPase requires the coordinated association of V_1_ subunits, which are synthesized in the cytosol, with V_O_ subunits, assembled in the ER. After association of the V_1_ subunits with membrane-bound V_O_ subunits, V_O_ subunit-bound ATP6AP2/PRR is partly cleaved by furin in the trans-Golgi network. Hence, the M8.9/CTF can be regarded as a remnant ATP6AP2/PRR rather than a functional protein, although we could not completely deny the possibility that a V-ATPase-bound CTF could direct subcellular targeting of the V-ATPase (Figure 7). Moreover, exogenous CTF expression appeared able to partially recover the reduced expression of V_O_ subunits and intravesicular acidification in ATP6AP2/PRR-deficient cells, indicating that M8.9/CTF certainly contains a sequence that is important, but still not sufficient to fully compensate for ATP6AP2/PRR-null MEFs.

Nevertheless, we still recommend caution in drawing conclusions from the present results based on the exogenous expression of truncated protein fragments. Exogenous expression of the CTF and NTF could be observed and seemed to reside in the expected cellular compartment, but we could not verify that the CTF and NTF were inserted and presented to the V-ATPase complex in the right manner. Because of the physical nature of the construct, such as lacking possible N-terminal signals for folding and membrane incorporation (not included in the signal peptide) and a C-terminal FLAG tag (which could cause unwanted effects in other systems), a negative result may not signal a lack of importance. Nevertheless, since exogenous full-length ATP6AP2/PRR successfully rescued the ATP6AP2/PRR-deficient cells, we believe that this study design was at least partially successful.

In this study, prorenin treatment did not affect the expression levels of V-ATPase subunits or the levels of intravesicular acidification. This was consistent with a previous study showing that renin had no effect on V-ATPase activity [14]. Regulation of V-ATPase activity is accomplished by a number of mechanisms, including reversible dissociation of V_1_V_O_ complexes, control of their cellular localization, and changes in the efficiency of coupling of proton transport with ATP hydrolysis [26]. Since we used LysoTracker staining to determine V-ATPase activity, we could not exclude the possibility that prorenin stimulation modestly alters V-ATPase activity. Further studies will be required to address this issue.

The yeast ortholog of furin, Kex2p, has been implicated in regulating V-ATPase activity [39]. V-ATPase activity is compromised in Kex2p mutant strains, although the protein is present in a fully assembled state, and thus potentially capable of full enzymatic activity [39]. The shedding of ATP6AP1/Ac45 by furin is important for V-ATPase-dependent intragranular acidification in the endocrine pancreas [40], and ATP6AP2/PRR is cleaved by furin in the trans-Golgi network [7]. Our study revealed that shedding of ATP6AP2/PRR by furin is dispensable for the function of V-ATPase, based upon the observations that neither overexpression nor downregulation of furin affected the protein expressions levels of V-ATPase subunits or the levels of intravesicular acidification, and that exogenously expressed furin-insensitive ATP6AP2/PRR mutant AKTA successfully rescued the ATP6AP2/PRR-null phenotype. This seems reasonable, since the assembly of Vo subunits is accomplished before reaching the trans-Golgi network, where shedding of ATP6AP2/PRR occurs. Since AKTA did not restore the expression levels of ATP6V0A1 and ATP6V0A2 sufficiently, the shedding of ATP6AP2/PRR would be important for targeting of the V-ATPase to subcellular organelles. Surprisingly, overexpression of furin did not increase the processing of ATP6AP2/PRR, suggesting the possible involvement of the other enzymes including ADAM19 in the cleavage of ATP6AP2/PRR [41], [42]. Future studies should elucidate how the cleavage of ATP6AP2/PRR is regulated in each tissue, as well as whether CTF cleaved from full-length ATP6AP2/PRR is still functional as a V-ATPase accessory protein or deficient, as observed with the exogenous expression of CTF in ATP6AP2/PRR-null cells.

The human Δ4M is known to be a cause of X-linked mental retardation Hedera type (MRXSH), in which patients a sysnonimous variant of *ATP6AP2*, c.321C>T (p.D107D) and a resultant markedly increased exon 4 skipping is identified [16]. Recently, another synonymous mutation in the same exon, c.345C>T (p.S115S), which also affects RNA splicing, was reported in X-linked parkinsonism with spasticity (XPDS) [15]. Previous reports demonstrated that human Δ4M could bind renin and increase renin catalytic activity, similar to full-length ATP6AP2/PRR, but exhibited a modest and reproducible impairment of prorenin- and renin-induced ERK1/2 activation [16], [17]. The structure of ATP6AP2/PRR is also evolutionally conserved among mammalian species [36]. In the current study, exogenously expressed human ATP6AP2/PRR successfully rescued the biogenesis of active murine V-ATPase in ATP6AP2/PRR-defective MEFs. On the other hand, human Δ4M failed to rescue the ATP6AP2/PRR-defective MEFs, suggesting an indispensable role of exon 4-encoded amino acids in the biogenesis of the active V-ATPase complex. Our study therefore showed, for the first time, that human Δ4M is defective in terms of ATP6AP2/PRR function as an accessory protein of the V-ATPase. That human Δ4M was obviously more unstable than other mutants of ATP6AP2/PRR could also explain the defects as a V-ATPase accessory protein. Based on these observations, reduction of the full-size ATP6AP2/PRR transcript and decreased level of ATP6AP2/PRR protein as well as deficient function of human Δ4M as a V-ATPase accessory protein in MRXSH and XPDS may compromise active V-ATPase biogenesis, and may ultimately be responsible for ATP6AP2/PRR haploinsufficiency as the underlying pathology. Further studies are thus necessary to elucidate the exact role of human Δ4M in the pathogenesis of MRXSH and XPDS.

ATP6AP2/PRR is a multi-functional protein; acting as a receptor for renin and prorenin, exerting RAS-related functions, associating with V-ATPase, and exerting fundamental cellular roles. The total ablation of ATP6AP2/PRR caused V-ATPase dysfunction and compromised intravesicular acidification. To test the physiological function of ATP6AP2/PRR as a receptor for prorenin and renin, it needs to rescue active V-ATPase biogenesis in ATP6AP2/PRR-null mice by transgenic overexpression of protein that functions as an accessory protein of V-ATPase, but lacks the ability to bind prorenin and renin. The results of our study indicated, however, that the NTF of ATP6AP2/PRR is required for both active V-ATPase biogenesis and binding with prorenin or renin. To design a construct that preserves the function of ATP6AP2/PRR as an accessory protein of V-ATPase, but abrogates the ability to bind prorenin and renin, requires resolution of the three dimensional structure to characterize precisely in molecular terms how prorenin or renin binds with ATP6AP2/PRR.

## Supporting Information

Figure S1 **Protein stability of V-ATPase subunits.** **A.** MEFs were treated in the presence or absence of ATP6AP2/PRR with cycloheximide (10 µM) to inhibit de novo protein synthesis and observed at various time points up to 6 h. Protein stability of V-ATPase subunits were unaffected even after ATP6AP2/PRR deletion (ATP6AP2Δ). **B.** Mouse and human full-length ATP6AP2/PRR were equally cleaved. Exogenous mouse and human full-sized ATP6AP2/PRR were transgenically overexpressed in MEFs, and immunoblot analysis was performed using anti-FLAG or anti-ATP6AP2/PRR antibody. mFL, mouse full-length ATP6AP2/PRR; hFL, human full-length ATP6AP2/PRR; CTF, carboxyl-terminal fragment; NTF, amino-terminal fragment.
(TIF)

Figure S2 **Protein stability of ATP6AP2/(pro)renin receptor (PRR) motifs.** **A.** Mouse embryonic fibroblasts (MEFs) were treated with cycloheximide (10 µM) to inhibit de novo protein synthesis 72 h after transfection with each FLAG-ATP6AP2/PRR mutant and observed at various time points up to 6 h. Immunoblot analysis using anti-FLAG and anti-ATP6AP2/PRR antibodies showed partial inhibition of the respective decreases in protein expression following proteasome inhibition with MG132 (5 µM). **B.** Half lives of each ATP6AP2/PRR construct were determined by immunoblot signal density quantification and calculating the approximate exponent curve. mFL, mouse full-length ATP6AP2/PRR; CTF, carboxyl-terminal fragment; CD, cytoplasmic domain; NTF, amino-terminal fragment; AKTA, ATP6AP2/PRR with mutagenesis in the potential furin cleavage R276A/KT/R279A site; hFL, human full-length ATP6AP2/PRR; hΔ4M, human ATP6AP2/PRR with a deletion of exon 4.
(TIF)

Figure S3 **Subcellular localization of endoplasmic reticulum and each ATP6AP2/(pro)renin receptor (PRR) motif.** **A.** MEFs overexpressing mutant ATP6AP2/PRRs were costained with anti-FLAG antibody and a marker for endoplasmic reticulum (BiP). **B.** The level of co-localization between BiP and FLAG was determined by Mander’s coefficients for colocalization. Scale bars, 20 µm. FL, full-length ATP6AP2/PRR; CTF, carboxyl-terminal fragment; CD, cytoplasmic domain; NTF, amino-terminal fragment; AKTA, ATP6AP2/PRR with mutagenesis in the potential furin cleavage R276A/KT/R279A site; hΔ4M, human ATP6AP2/PRR with a deletion of exon 4; R, Mander’s overlap coefficient; Mbip, Mander’s colocalization coefficient for BiP; Mflag, Mander’s colocalization coefficient for FLAG.
(TIF)

Figure S4 **Subcellular localization of Golgi and each ATP6AP2/(pro)renin receptor (PRR) motif.** **A.** MEFs overexpressing mutant ATP6AP2/PRRs were costained with anti-FLAG antibody and a marker for Golgi (GM130). **B.** The level of colocalization between GM130 and FLAG was determined by Mander’s coefficients for colocalization. Scale bars, 10 µm (the third panels from the left), 20 µm (other panels). FL, full-length ATP6AP2/PRR; CTF, carboxyl-terminal fragment; CD, cytoplasmic domain; NTF, amino-terminal fragment; AKTA, ATP6AP2/PRR with mutagenesis in the potential furin cleavage R276A/KT/R279A site; hΔ4M, human ATP6AP2/PRR with a deletion of exon 4; R, Mander’s overlap coefficient; Mgm130, Mander’s colocalization coefficient for GM130; Mflag, Mander’s colocalization coefficient for FLAG.
(TIF)

Figure S5 **Subcellular localization of early endosomes (EEA1) and each ATP6AP2/(pro)renin receptor (PRR) motif.** **A.** MEFs overexpressing mutant ATP6AP2/PRRs were costained with anti-FLAG antibody and a marker for early endosomes (EEA1). **B.** The level of colocalization between EEA1 and FLAG was determined by Mander’s coefficients for colocalization. Scale bars, 20 µm. FL, full-length ATP6AP2/PRR; CTF, carboxyl-terminal fragment; CD, cytoplasmic domain; NTF, amino-terminal fragment; AKTA, ATP6AP2/PRR with mutagenesis in the potential furin cleavage R276A/KT/R279A site; hΔ4M, human ATP6AP2/PRR with a deletion of exon 4; R, Mander’s overlap coefficient; Meea1, Mander’s colocalization coefficient for EEA1; Mflag, Mander’s colocalization coefficient for FLAG.
(TIF)

Figure S6 **Subcellular localization of early endosomes (Rab5) and each ATP6AP2/(pro)renin receptor (PRR) motif.** **A.** MEFs overexpressing mutant ATP6AP2/PRRs were costained with anti-FLAG antibody and a marker for early endosomes (Rab5). **B.** The level of colocalization between Rab5 and FLAG was determined by Mander’s coefficients for colocalization. Scale bars, 20 µm. FL, full-length ATP6AP2/PRR; CTF, carboxyl-terminal fragment; CD, cytoplasmic domain; NTF, amino-terminal fragment; AKTA, ATP6AP2/PRR with mutagenesis in the potential furin cleavage R276A/KT/R279A site; hΔ4M, human ATP6AP2/PRR with a deletion of exon 4; R, Mander’s overlap coefficient; Mrab5, Mander’s colocalization coefficient for Rab5; Mflag, Mander’s colocalization coefficient for FLAG.
(TIF)

Figure S7 **Subcellular localization of late endosomes and each ATP6AP2/(pro)renin receptor (PRR) motif.** **A.** MEFs overexpressing mutant ATP6AP2/PRRs were costained with anti-FLAG antibody and a marker for late endosomes (Rab7). **B.** The level of colocalization between Rab7 and FLAG was determined by Mander’s coefficients for colocalization. Scale bars, 20 µm. FL, full-length ATP6AP2/PRR; CTF, carboxyl-terminal fragment; CD, cytoplasmic domain; NTF, amino-terminal fragment; AKTA, ATP6AP2/PRR with mutagenesis in the potential furin cleavage R276A/KT/R279A site; hΔ4M, human ATP6AP2/PRR with a deletion of exon 4; R, Mander’s overlap coefficient; Mrab7, Mander’s colocalization coefficient for Rab7; Mflag, Mander’s colocalization coefficient for FLAG.
(TIF)

Figure S8 **Subcellular localization of lysosomes and each ATP6AP2/(pro)renin receptor (PRR) motif.** **A.** MEFs overexpressing mutant ATP6AP2/PRRs were costained with anti-FLAG antibody and a marker for lysosomes (Lamp2). **B.** The level of colocalization between Lamp2 and FLAG was determined by Mander’s coefficients for colocalization. Scale bars, 20 µm. FL, full-length ATP6AP2/PRR; CTF, carboxyl-terminal fragment; CD, cytoplasmic domain; NTF, amino-terminal fragment; AKTA, ATP6AP2/PRR with mutagenesis in the potential furin cleavage R276A/KT/R279A site; hΔ4M, human ATP6AP2/PRR with a deletion of exon 4; R, Mander’s overlap coefficient; Mlamp2, Mander’s colocalization coefficient for Lamp2; Mflag, Mander’s colocalization coefficient for FLAG.
(TIF)

Figure S9 **Subcellular localization of V-ATPase and each ATP6AP2/(pro)renin receptor (PRR) motif.** **A.** MEFs overexpressing mutant ATP6AP2/PRRs were costained with anti-FLAG antibody and a marker for ATP6V0C. **B.** The level of colocalization between ATP6V0C and FLAG was determined by Mander’s coefficients for colocalization. Scale bars, 10 µm (the second panels from the left), 20 µm (other panels). FL, full-length ATP6AP2/PRR; CTF, carboxyl-terminal fragment; CD, cytoplasmic domain; NTF, amino-terminal fragment; AKTA, ATP6AP2/PRR with mutagenesis in the potential furin cleavage R276A/KT/R279A site; hΔ4M, human ATP6AP2/PRR with a deletion of exon 4; R, Mander’s overlap coefficient; Matp6v0c, Mander’s colocalization coefficient for ATP6V0C; Mflag, Mander’s colocalization coefficient for FLAG.
(TIF)

Figure S10 **Binding of prorenin to ATP6AP2/PRR had no effect on the biogenesis of active V-ATPase.** **A,** Wild-type MEFs were subject to 2 nM prorenin. Prorenin did not affect expression levels of V-ATPase subunits, although prorenin stimulation increased the phosphorylation levels of ERK1/2. **B,** LysoTracker staining demonstrated that prorenin did not influence intravesicular acidification. **C,** Quantitative analysis of protein expressions of ATP6V0A1, ATP6V0A2, ATP6V0A3, ATP6V0C, and ATP6V1E2 in MEFs. The data are presented as the ratio of protein expressions in 60 min to those in 0 min. Graphed data show the mean ± SD. FL, full-length ATP6AP2/PRR; NTF, amino-terminal fragment; LT, LysoTracker. Scale bar: 20 µm.
(TIF)
