# Supplementary figures and images for: The Role of Individual Domains and the Significance of Shedding of ATP6AP2/(pro)renin Receptor in Vacuolar H+-ATPase Biogenesis
Source: PLoS One. 2013 Nov 4;8(11):e78603. doi: 10.1371/journal.pone.0078603 (PMC3817224; doi:10.1371/journal.pone.0078603)

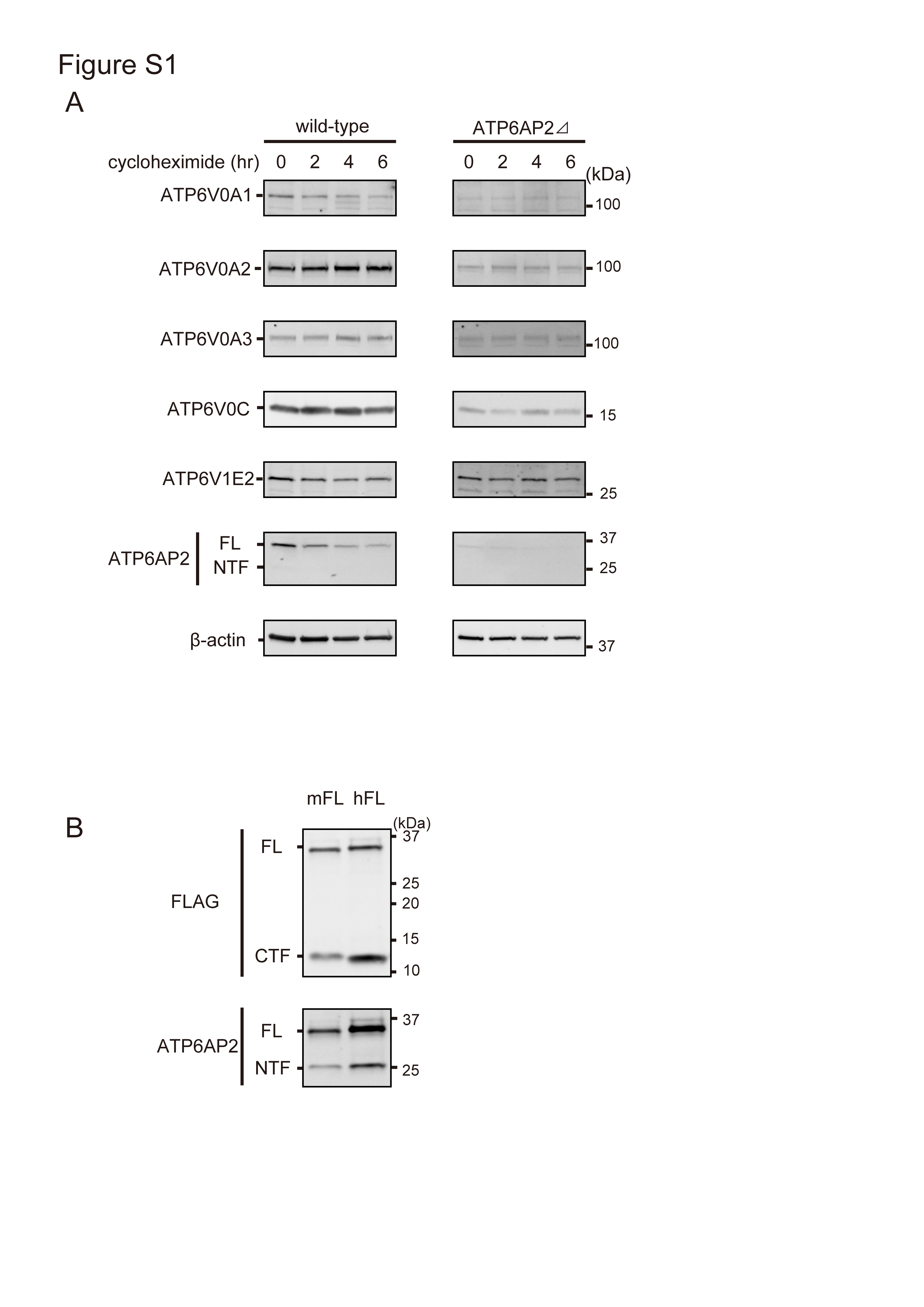

Supplement: Figure S1 — Protein stability of V-ATPase subunits. A. MEFs were treated in the presence or absence of ATP6AP2/PRR with cycloheximide (10 µM) to inhibit de novo protein synthesis and observed at various time points up to 6 h. Protein stability of V-ATPase subunits were unaffected even after ATP6AP2/PRR deletion (ATP6AP2Δ). B. Mouse and human full-length ATP6AP2/PRR were equally cleaved. Exogenous mouse and human full-sized ATP6AP2/PRR were transgenically overexpressed in MEFs, and immunoblot analysis was performed using anti-FLAG or anti-ATP6AP2/PRR antibody. mFL, mouse full-length ATP6AP2/PRR; hFL, human full-length ATP6AP2/PRR; CTF, carboxyl-terminal fragment; NTF, amino-terminal fragment. (TIF) [file pone.0078603.s001.tif]

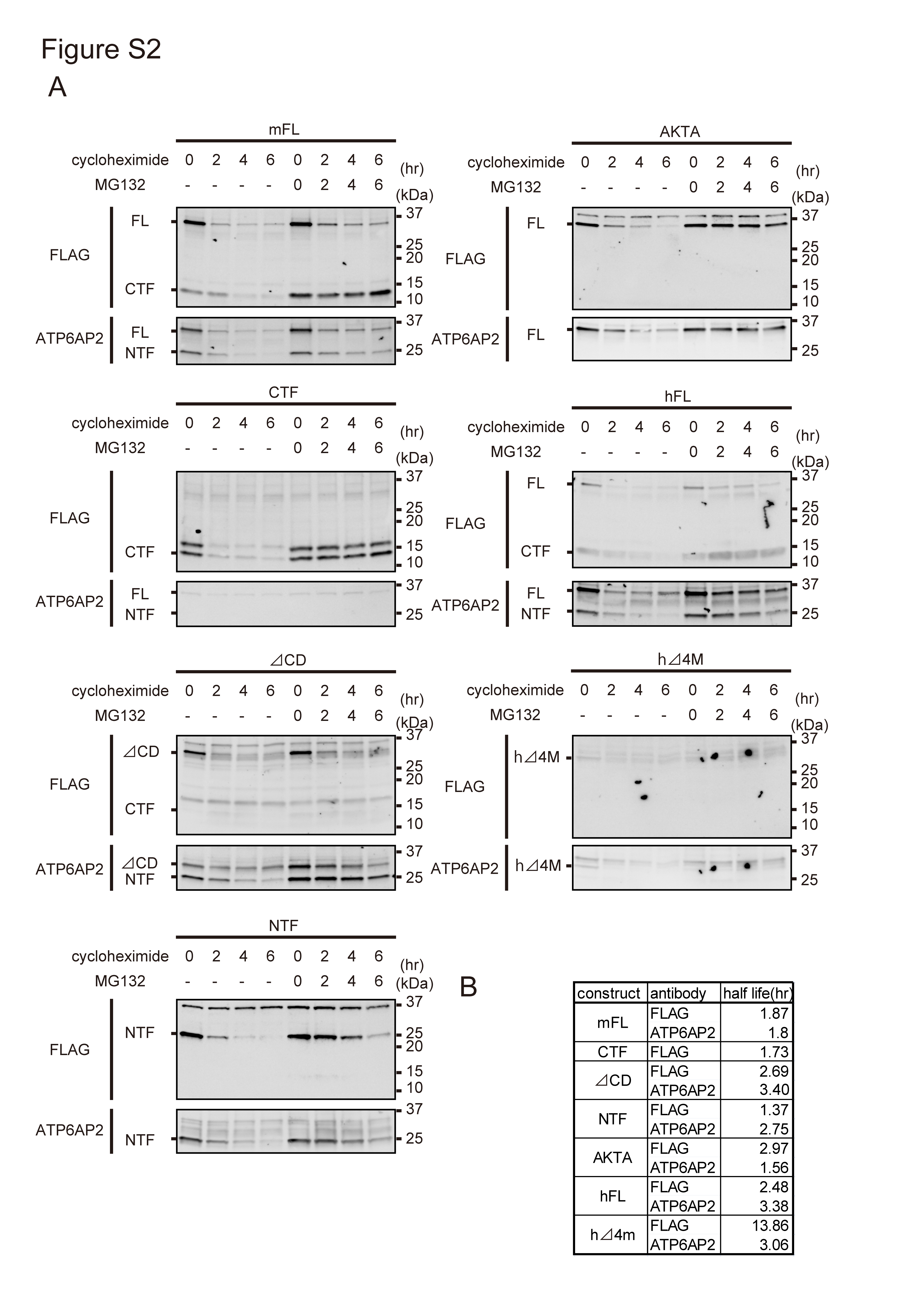

Supplement: Figure S2 — Protein stability of ATP6AP2/(pro)renin receptor (PRR) motifs. A. Mouse embryonic fibroblasts (MEFs) were treated with cycloheximide (10 µM) to inhibit de novo protein synthesis 72 h after transfection with each FLAG-ATP6AP2/PRR mutant and observed at various time points up to 6 h. Immunoblot analysis using anti-FLAG and anti-ATP6AP2/PRR antibodies showed partial inhibition of the respective decreases in protein expression following proteasome inhibition with MG132 (5 µM). B. Half lives of each ATP6AP2/PRR construct were determined by immunoblot signal density quantification and calculating the approximate exponent curve. mFL, mouse full-length ATP6AP2/PRR; CTF, carboxyl-terminal fragment; CD, cytoplasmic domain; NTF, amino-terminal fragment; AKTA, ATP6AP2/PRR with mutagenesis in the potential furin cleavage R276A/KT/R279A site; hFL, human full-length ATP6AP2/PRR; hΔ4M, human ATP6AP2/PRR with a deletion of exon 4. (TIF) [file pone.0078603.s002.tif]

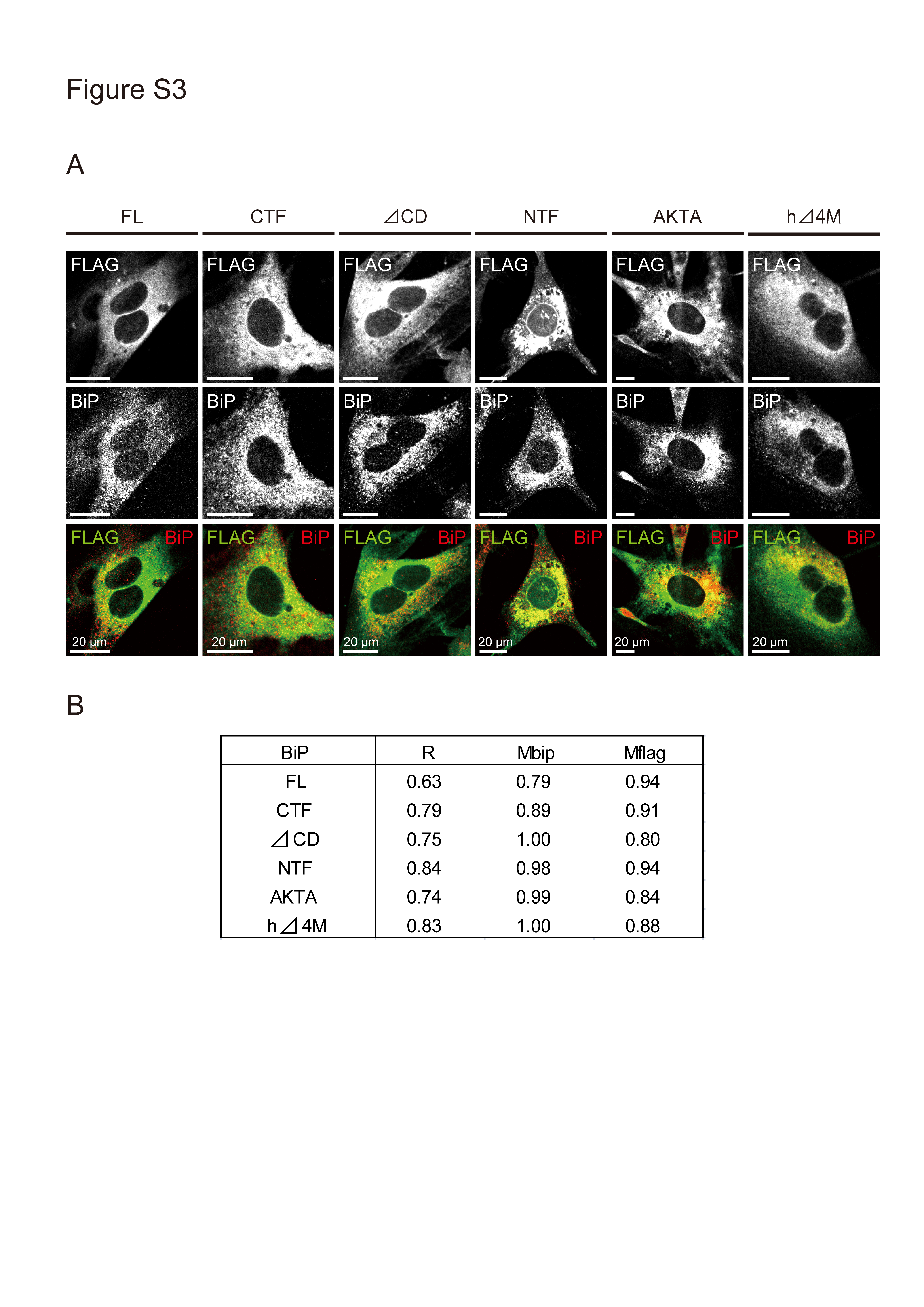

Supplement: Figure S3 — Subcellular localization of endoplasmic reticulum and each ATP6AP2/(pro)renin receptor (PRR) motif. A. MEFs overexpressing mutant ATP6AP2/PRRs were costained with anti-FLAG antibody and a marker for endoplasmic reticulum (BiP). B. The level of co-localization between BiP and FLAG was determined by Mander’s coefficients for colocalization. Scale bars, 20 µm. FL, full-length ATP6AP2/PRR; CTF, carboxyl-terminal fragment; CD, cytoplasmic domain; NTF, amino-terminal fragment; AKTA, ATP6AP2/PRR with mutagenesis in the potential furin cleavage R276A/KT/R279A site; hΔ4M, human ATP6AP2/PRR with a deletion of exon 4; R, Mander’s overlap coefficient; Mbip, Mander’s colocalization coefficient for BiP; Mflag, Mander’s colocalization coefficient for FLAG. (TIF) [file pone.0078603.s003.tif]

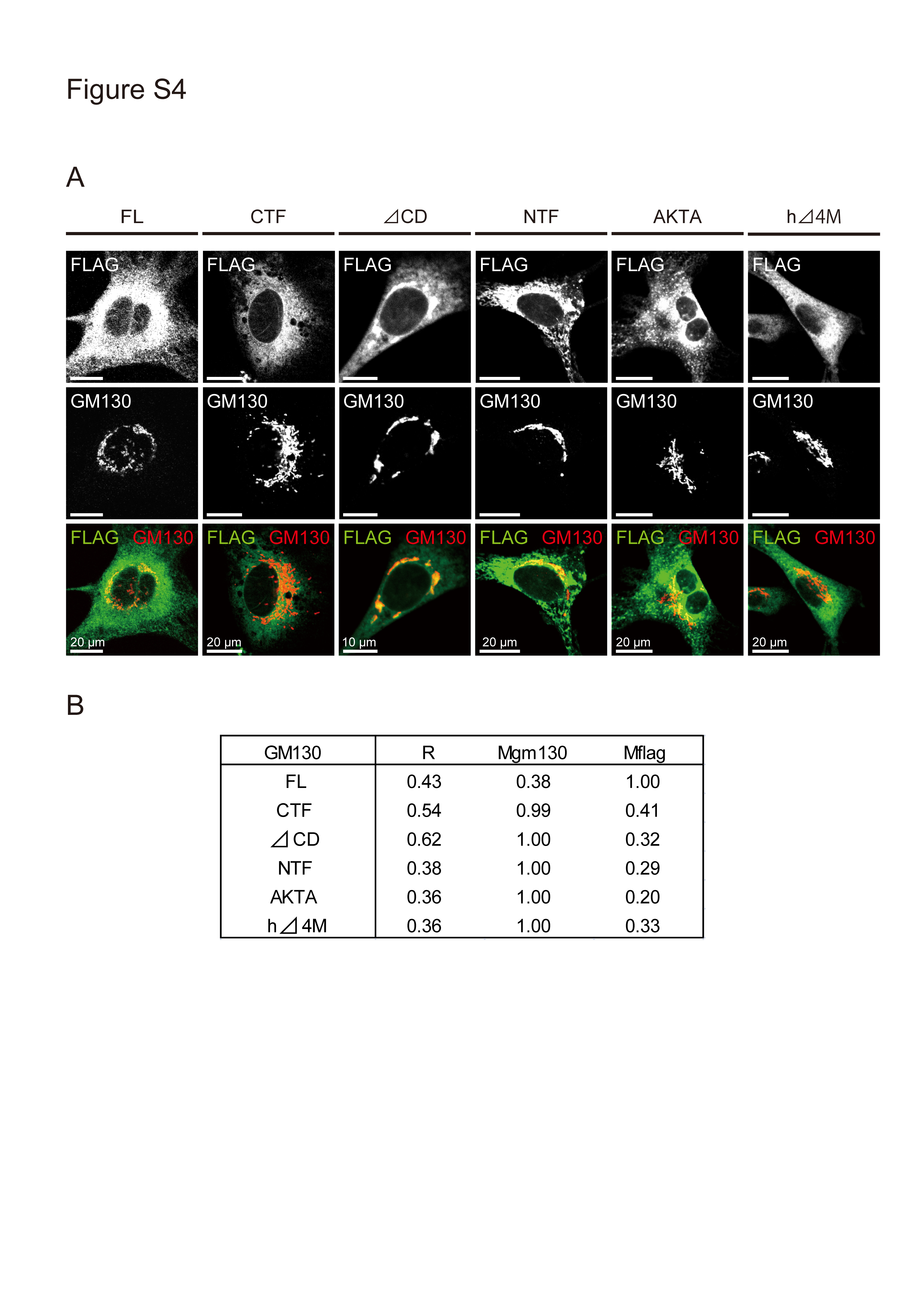

Supplement: Figure S4 — Subcellular localization of Golgi and each ATP6AP2/(pro)renin receptor (PRR) motif. A. MEFs overexpressing mutant ATP6AP2/PRRs were costained with anti-FLAG antibody and a marker for Golgi (GM130). B. The level of colocalization between GM130 and FLAG was determined by Mander’s coefficients for colocalization. Scale bars, 10 µm (the third panels from the left), 20 µm (other panels). FL, full-length ATP6AP2/PRR; CTF, carboxyl-terminal fragment; CD, cytoplasmic domain; NTF, amino-terminal fragment; AKTA, ATP6AP2/PRR with mutagenesis in the potential furin cleavage R276A/KT/R279A site; hΔ4M, human ATP6AP2/PRR with a deletion of exon 4; R, Mander’s overlap coefficient; Mgm130, Mander’s colocalization coefficient for GM130; Mflag, Mander’s colocalization coefficient for FLAG. (TIF) [file pone.0078603.s004.tif]

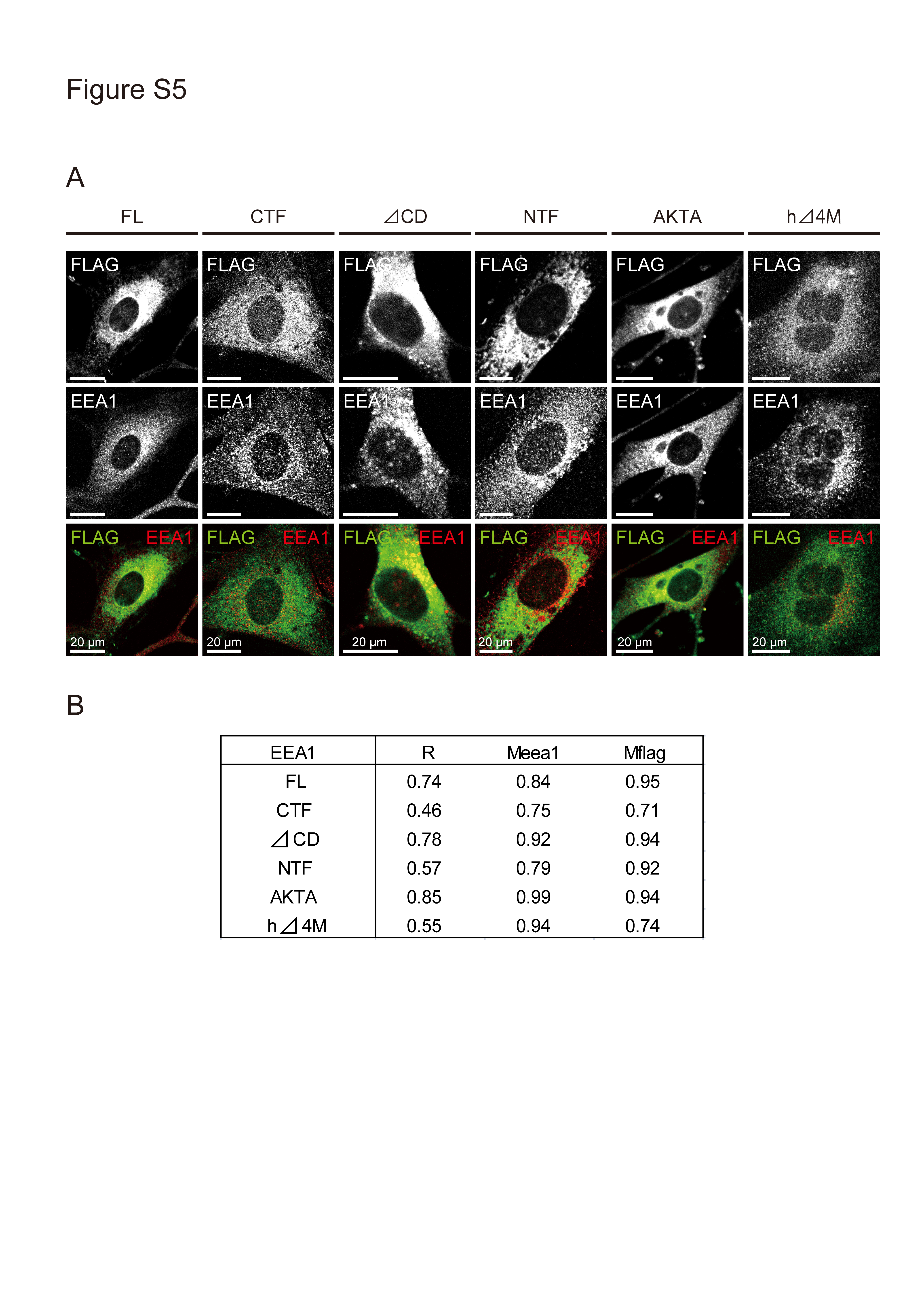

Supplement: Figure S5 — Subcellular localization of early endosomes (EEA1) and each ATP6AP2/(pro)renin receptor (PRR) motif. A. MEFs overexpressing mutant ATP6AP2/PRRs were costained with anti-FLAG antibody and a marker for early endosomes (EEA1). B. The level of colocalization between EEA1 and FLAG was determined by Mander’s coefficients for colocalization. Scale bars, 20 µm. FL, full-length ATP6AP2/PRR; CTF, carboxyl-terminal fragment; CD, cytoplasmic domain; NTF, amino-terminal fragment; AKTA, ATP6AP2/PRR with mutagenesis in the potential furin cleavage R276A/KT/R279A site; hΔ4M, human ATP6AP2/PRR with a deletion of exon 4; R, Mander’s overlap coefficient; Meea1, Mander’s colocalization coefficient for EEA1; Mflag, Mander’s colocalization coefficient for FLAG. (TIF) [file pone.0078603.s005.tif]

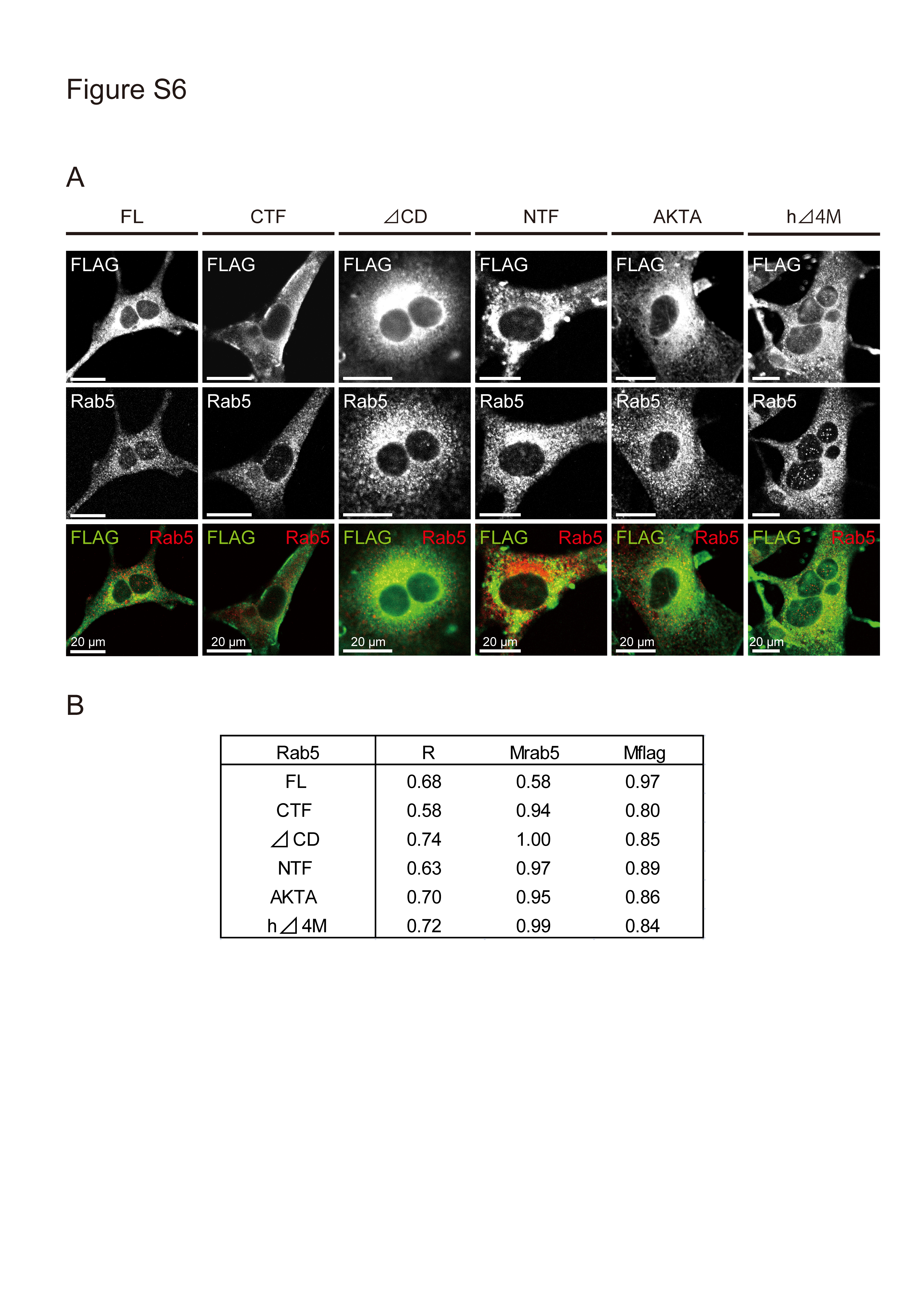

Supplement: Figure S6 — Subcellular localization of early endosomes (Rab5) and each ATP6AP2/(pro)renin receptor (PRR) motif. A. MEFs overexpressing mutant ATP6AP2/PRRs were costained with anti-FLAG antibody and a marker for early endosomes (Rab5). B. The level of colocalization between Rab5 and FLAG was determined by Mander’s coefficients for colocalization. Scale bars, 20 µm. FL, full-length ATP6AP2/PRR; CTF, carboxyl-terminal fragment; CD, cytoplasmic domain; NTF, amino-terminal fragment; AKTA, ATP6AP2/PRR with mutagenesis in the potential furin cleavage R276A/KT/R279A site; hΔ4M, human ATP6AP2/PRR with a deletion of exon 4; R, Mander’s overlap coefficient; Mrab5, Mander’s colocalization coefficient for Rab5; Mflag, Mander’s colocalization coefficient for FLAG. (TIF) [file pone.0078603.s006.tif]

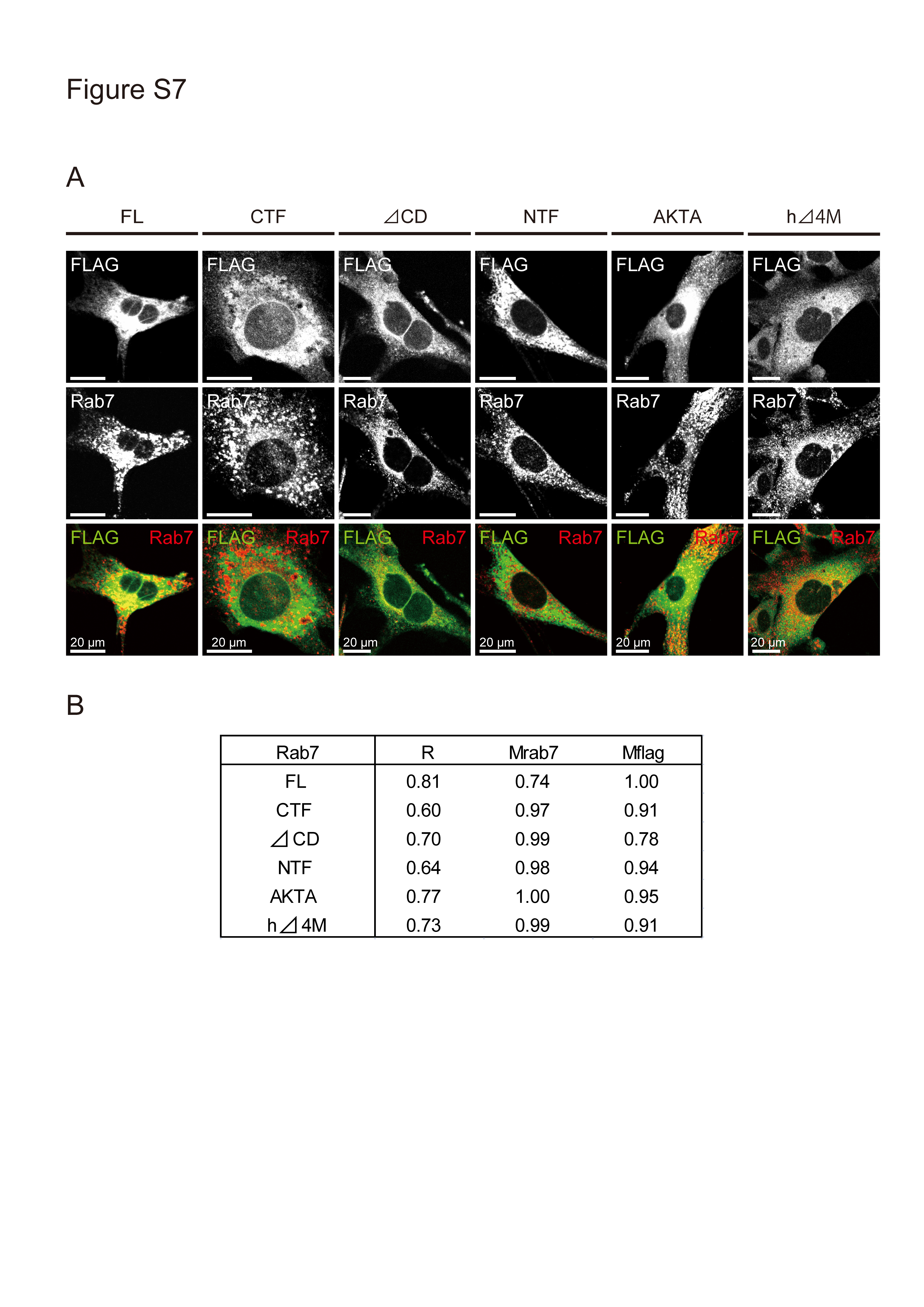

Supplement: Figure S7 — Subcellular localization of late endosomes and each ATP6AP2/(pro)renin receptor (PRR) motif. A. MEFs overexpressing mutant ATP6AP2/PRRs were costained with anti-FLAG antibody and a marker for late endosomes (Rab7). B. The level of colocalization between Rab7 and FLAG was determined by Mander’s coefficients for colocalization. Scale bars, 20 µm. FL, full-length ATP6AP2/PRR; CTF, carboxyl-terminal fragment; CD, cytoplasmic domain; NTF, amino-terminal fragment; AKTA, ATP6AP2/PRR with mutagenesis in the potential furin cleavage R276A/KT/R279A site; hΔ4M, human ATP6AP2/PRR with a deletion of exon 4; R, Mander’s overlap coefficient; Mrab7, Mander’s colocalization coefficient for Rab7; Mflag, Mander’s colocalization coefficient for FLAG. (TIF) [file pone.0078603.s007.tif]

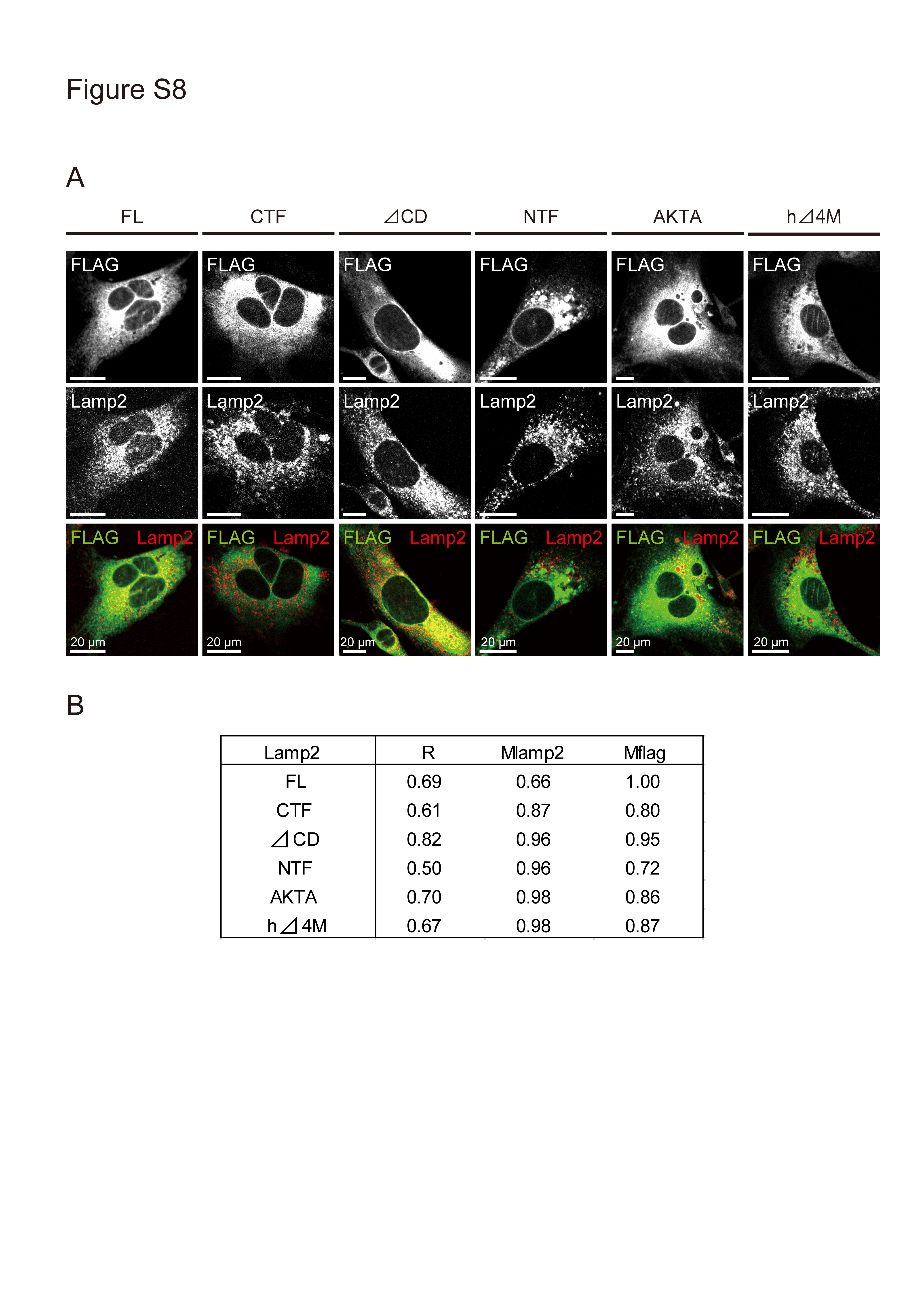

Supplement: Figure S8 — Subcellular localization of lysosomes and each ATP6AP2/(pro)renin receptor (PRR) motif. A. MEFs overexpressing mutant ATP6AP2/PRRs were costained with anti-FLAG antibody and a marker for lysosomes (Lamp2). B. The level of colocalization between Lamp2 and FLAG was determined by Mander’s coefficients for colocalization. Scale bars, 20 µm. FL, full-length ATP6AP2/PRR; CTF, carboxyl-terminal fragment; CD, cytoplasmic domain; NTF, amino-terminal fragment; AKTA, ATP6AP2/PRR with mutagenesis in the potential furin cleavage R276A/KT/R279A site; hΔ4M, human ATP6AP2/PRR with a deletion of exon 4; R, Mander’s overlap coefficient; Mlamp2, Mander’s colocalization coefficient for Lamp2; Mflag, Mander’s colocalization coefficient for FLAG. (TIF) [file pone.0078603.s008.tif]

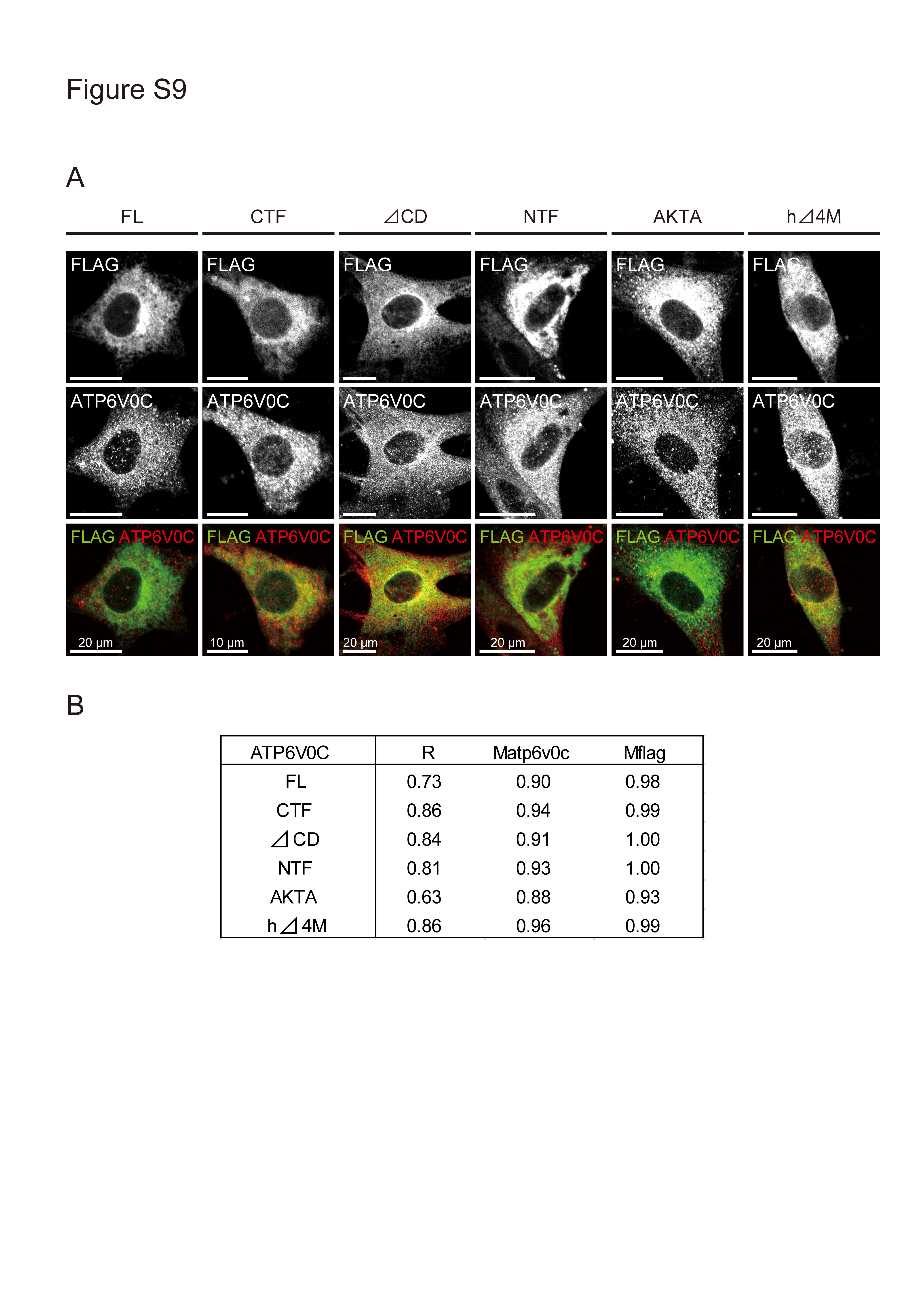

Supplement: Figure S9 — Subcellular localization of V-ATPase and each ATP6AP2/(pro)renin receptor (PRR) motif. A. MEFs overexpressing mutant ATP6AP2/PRRs were costained with anti-FLAG antibody and a marker for ATP6V0C. B. The level of colocalization between ATP6V0C and FLAG was determined by Mander’s coefficients for colocalization. Scale bars, 10 µm (the second panels from the left), 20 µm (other panels). FL, full-length ATP6AP2/PRR; CTF, carboxyl-terminal fragment; CD, cytoplasmic domain; NTF, amino-terminal fragment; AKTA, ATP6AP2/PRR with mutagenesis in the potential furin cleavage R276A/KT/R279A site; hΔ4M, human ATP6AP2/PRR with a deletion of exon 4; R, Mander’s overlap coefficient; Matp6v0c, Mander’s colocalization coefficient for ATP6V0C; Mflag, Mander’s colocalization coefficient for FLAG. (TIF) [file pone.0078603.s009.tif]

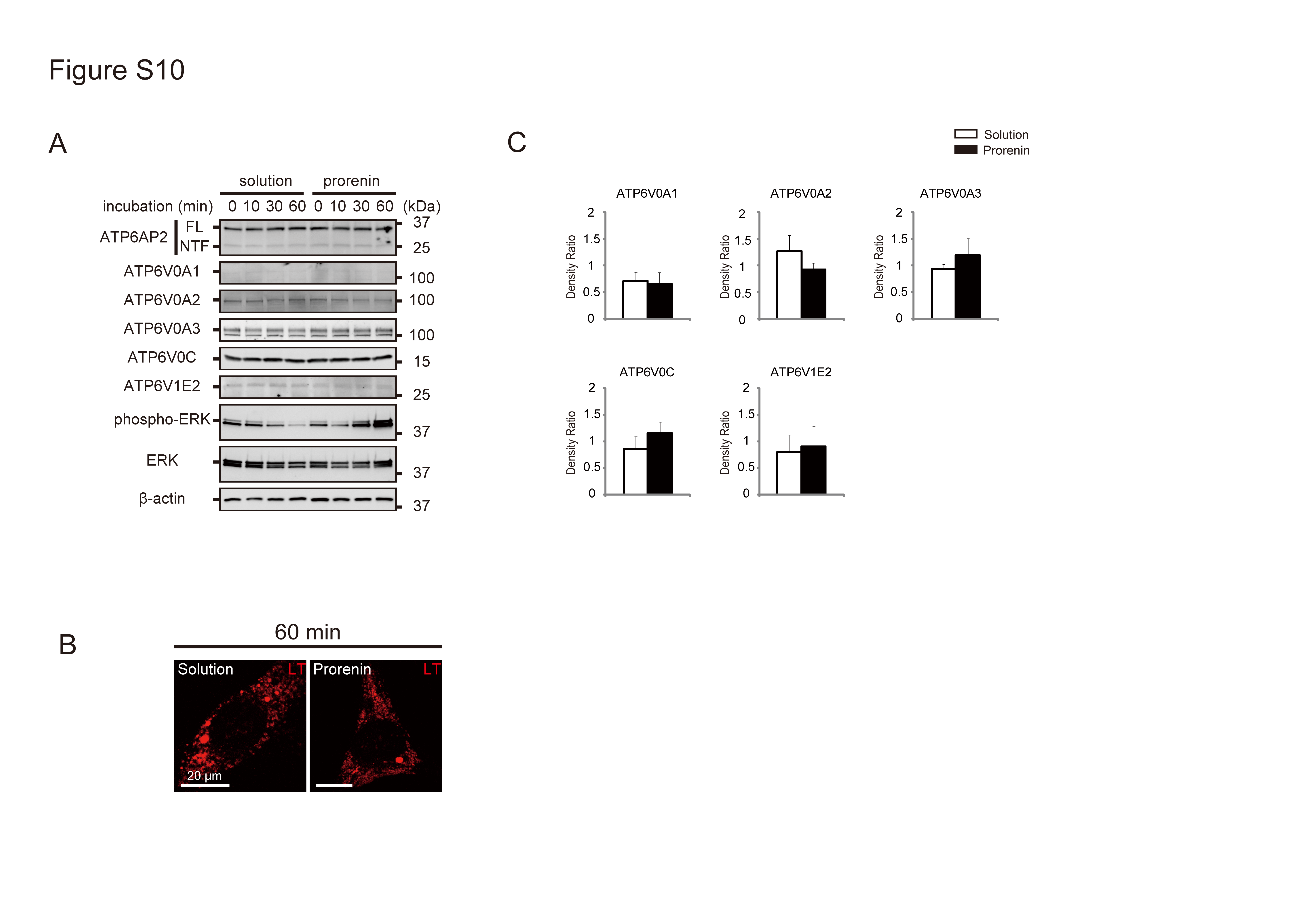

Supplement: Figure S10 — Binding of prorenin to ATP6AP2/PRR had no effect on the biogenesis of active V-ATPase. A, Wild-type MEFs were subject to 2 nM prorenin. Prorenin did not affect expression levels of V-ATPase subunits, although prorenin stimulation increased the phosphorylation levels of ERK1/2. B, LysoTracker staining demonstrated that prorenin did not influence intravesicular acidification. C, Quantitative analysis of protein expressions of ATP6V0A1, ATP6V0A2, ATP6V0A3, ATP6V0C, and ATP6V1E2 in MEFs. The data are presented as the ratio of protein expressions in 60 min to those in 0 min. Graphed data show the mean ± SD. FL, full-length ATP6AP2/PRR; NTF, amino-terminal fragment; LT, LysoTracker. Scale bar: 20 µm. (TIF) [file pone.0078603.s010.tif]
